# A clinical and mechanistic study on traditional Chinese medicine herbs used in olfactory training

**DOI:** 10.1186/s13020-026-01432-x

**Published:** 2026-06-02

**Authors:** Wei Zhang, Yingjian Liu, Jiahui Sun, Fei Wang, Licong Nie, Jinxin Chen, Hang Ren, Jiaxin Chen, Yu Zheng, Weihong Liu, Yonggang Liu

**Affiliations:** 1 Otolaryngology Department, Eye Hospital China Academy of Chinese Medical Science, Beijing, 100040 China; 2 Hanwang Technology Co., Ltd, Beijing, 100193 China; 3 Intelligent Perception Laboratory, Beijing, 100193 China

**Keywords:** OD, olfactory dysfunction, OT, olfactory training, TCM, traditional Chinese medicine, Herb, OR, olfactory receptor, SAR, structure–activity relationship

## Abstract

**Background:**

Olfactory dysfunction (OD) is a prevalent condition with limited treatment options, severely impairing patients’ quality of life. Olfactory training (OT) using conventional odorants provides limited benefit. Traditional Chinese Medicine (TCM) herbs with complex odors may offer novel OT agents, but their mechanism and clinical value remain unclear.

**Methods:**

In a prospective, open-label, parallel-controlled clinical observation, 73 OD patients (TCM-OT group: n = 36; control group: n = 37) received either 3-month TCM-based OT (6 herbs) or mometasone furoate nasal spray. Olfactory function was assessed by olfactory identification test (OIT), visual analog scale (VAS), and quality-of-life questionnaire (QOD). Volatile profiles of the six herbs were characterized by GC-MS. We then performed systematic functional screening of 16 major herb odorants and 4 common chemical odorants (eugenol, citronellal, phenylethyl alcohol, eucalyptol) against ~ 400 human olfactory receptors (hORs) using luciferase assays. Results were combined with public database entries (M2OR, OlfactionBase) to compare OR activation patterns. Molecular docking and molecular dynamics (MD) simulations were performed for (-)-Borneol, a representative active component, against its responsive ORs.

**Results:**

TCM-OT significantly improved the primary outcome (OIT) compared with control (mean improvement 11.31 ± 6.30 vs. 6.22 ± 4.99, p < 0.001, Cohen’s d = 1.150). Secondary outcomes (VAS and QOD) showed trends favoring TCM-OT but did not reach statistical significance after Bonferroni correction. Greater benefit was observed in younger patients. Herb odorants displayed higher chemical diversity than chemical odorants. Functional screening identified 34 odorant-OR activation pairs for herb odorants (23 novel) and 11 pairs for chemical odorants (10 novel). Integrated analysis revealed that herb odorants collectively activated all 17 hOR families, whereas chemical odorants engaged only 8 families. Molecular simulations further elucidated the multi-receptor recognition mechanism of (-)-Borneol.

**Conclusions:**

TCM-OT is a promising multi-target approach for OD. The broad activation of all OR families by herb odorants — contrasting with the limited engagement of conventional chemical odorants — provides a molecular rationale for the efficacy of TCM-OT and guidance for the selection of olfactory agents used in OT.

**Trial registration:**

This study was retrospectively registered with the Eye Hospital of China Academy of Chinese Medical Sciences (YKEC-KT-2025-057-P002).

**Supplementary Information:**

The online version contains supplementary material available at 10.1186/s13020-026-01432-x.

## Introduction

Olfactory dysfunction (OD) represents a complex and often overlooked clinical challenge affecting approximately 20% of the general population, with prevalence increasing markedly with age [1, 2]. OD encompasses a spectrum of disorders including anosmia (complete loss of smell), hyposmia (reduced smell sensitivity), parosmia (distorted odor perception), and phantosmia (odor perception without an external stimulus) [3, 4]. The functional implications of OD extend beyond diminished sensory pleasure, significantly impacting nutrition, hazard detection, social interactions, and psychological well-being [5]. The COVID-19 pandemic has further highlighted the clinical importance of OD, with a substantial proportion of patients experiencing persistent olfactory deficits as a hallmark of post-COVID syndrome [6].

The pathophysiological mechanisms underlying OD are diverse and multifaceted. Conductive OD results from obstruction of the nasal passages preventing odorant access to the olfactory epithelium, while sensorineural OD involves damage to the olfactory neuroepithelium itself [7–9]. These pathologies frequently stem from several key etiologies, including infection, aging, and neurodegeneration [10]. More recently, genetic factors have also been identified as significant contributors to olfactory function variability [11].

The complexity and heterogeneity of these mechanisms have considerably challenged the development of effective treatments, rendering many current management strategies of limited or variable efficacy. Current clinical management includes olfactory training (OT), corticosteroid therapy, nutritional supplementation, and, in select cases, surgical intervention [12, 13]. However, invasive options such as surgery often produce suboptimal and unpredictable outcomes [14], highlighting the need for safer and more reliable noninvasive alternatives. Against this background, OT has emerged as a particularly promising noninvasive approach, leveraging the neuroplasticity of the olfactory system to drive functional recovery through repeated exposure to standardized odorants [15]. The seminal work by Hummel et al. first demonstrated that structured short-term exposure to 4 specific odorants (phenylethyl alcohol, eucalyptol, citronellal, and eugenol) over 12 weeks significantly improved olfactory function in approximately 30% of patients with various causes of olfactory loss [16]. This pioneering study established OT as a viable therapeutic option and inspired subsequent research.

The mechanistic basis of OT appears to involve modulation of the olfactory system at multiple levels. In humans, neuroimaging and electrophysiological studies suggest that OT promotes functional and structural neuroplasticity, extending beyond the peripheral sensory epithelium to central brain regions involved in odor discrimination and memory, such as the olfactory bulb and piriform cortex [15]. Animal studies have demonstrated that repeated odor exposure can enhance the regeneration of olfactory receptor neurons and increase the expression of olfactory receptors (ORs) [17–19].

The enhanced OR expression and neuroplasticity induced by OT fundamentally rely on the molecular activation of ORs. This process is governed by a combinatorial coding scheme: a single odorant can activate multiple ORs, and a single OR can be activated by multiple odorants [20, 21]. Recent advances in OR deorphanization techniques and the accumulation of OR-ligand interaction data in public databases now enable more comprehensive analysis of odorant-OR relationships [22, 23]. Furthermore, structural biology and computational approaches have provided unprecedented insights into the molecular recognition mechanisms governing OR activation, revealing how structurally diverse compounds can interact with overlapping yet distinct OR ensembles [24–27].

Traditional Chinese medicine (TCM) has a long history of application in the treatment of olfactory disorders, and contemporary clinical research is progressively validating its efficacy [28–30]. Similar to established odors such as lemon, rose, eucalyptus, and clove, certain TCM herbs exhibit distinct and potent odors, suggesting their potential as novel agents for olfactory training [28]. Modern analytical techniques, particularly gas chromatography-mass spectrometry (GC-MS), are now enabling the systematic characterization of the complex volatile profiles of these herbs, thereby facilitating the correlation between their specific chemical components and olfactory activity [31–33].

This study enrolled the TCM-OT group between October 2023 and February 2024, while control group recruitment proceeded more gradually; with continued enrollment, both cohorts achieved adequate size for comparison, yielding a final sample of 36 patients in the TCM-OT group and 37 patients in the control group. Our integrated approach first confirmed the clinical efficacy of TCM-OT and then systematically decoded the chemical profiles of the six herbs used. We mapped the activation patterns of TCM herb odorants through large-scale functional screening against approximately 400 human ORs, comparing them with conventional chemical odorants. Furthermore, molecular docking and dynamics simulations were employed to unravel the structural basis of multi-OR recognition by a key herbal component.

This research explores the potential of TCM formulations as a complementary approach for olfactory rehabilitation, pending validation in larger randomized controlled trials. By elucidating the underlying mechanisms, our work provides preliminary evidence and mechanistic insights that may inform future evidence-based strategies to advance OD rehabilitation practice.

## Methods

### Trial design

This was a single-center, prospective, open-abel clinical observation. The final analysis included 73 patients who completed the 3-month treatment protocol: 36 in the herb group (TCM-OT) and 37 in the control group (mometasone furoate nasal spray). A pre-post design with between-group comparisons was used to assess olfactory function before and after the intervention.

### TCM herbs and nasal spray

The selection of six TCM herbs (Borneol, Caryophylli Flos, Dried Ginger, Pogostemon Cablin, Myristica Fragrans, and Magnoliae Flos) was based on three key principles: (1) Classic TCM theory of "aromatic orifice-opening", supported by Lingshu Maidu which states that lung qi connects to the nose and aromatic herbs can restore olfactory function; (2) Classical prescription foundation, particularly drawing from Xinyi San and Cangerzi San which historically use Magnoliae Flos and Caryophylli Flos for nasal disorder, with Borneol opening orifices and awakening the spirit, Dried Ginger warming the lung and transforming phlegm, Pogostemon Cablin aromatically transforming dampness, and Myristica Fragrans warming the spleen and stomach; and (3) Clinincal validation through our preliminary practice demonstrating good efficacy and tolerance using an inhalation-first approach, which represents the clinical application of Wu Shiji's nasal insufflation method. All herbs were individually ground into a fine powder using a High-Speed Multifunction Grinder (Model 800Y) and sieved to ensure uniformity. For OT, 5 g of each herbal powder was packaged in separate, breathable sachets. The control group was treated with mometasone furoate nasal spray (Schering-Plough).

### Clinical OT method

This prospective study enrolled eligible patients with OD from the Otolaryngology outpatient clinic of the Eye Hospital, China Academy of Chinese Medical Sciences. The studies involving human participants were reviewed and approved by the Committee of Medical Ethics of Eye Hospital China Academy of Chinese Medical Science (YKEC-KT-2025-057-P002). Written Informed Consent was obtained from the patients.

Herb group: Between October 2023 and February 2024, 62 patients who met the diagnostic and inclusion criteria were recruited for TCM-OT. Of these, 36 completed the full 12-week study protocol and were included in the final analysis (dropout rate: 41.9%).

Control group: Recruitment for mometasone furoate nasal spray treatment proceeded over an extended period until April 2026, with 41 patients enrolled and 37 completing the 12-week study protocol (dropout rate: 9.8%).

Final analysis: A total of 73 patients were included in the final analysis (36 in herb group, 37 in control group).

Inclusion Criteria: (1) Aged 18-75 years; (2) Diagnosed with olfactory dysfunction by nasal endoscopy, imaging examination, and olfactory function test, with a confirmed etiology of post-infectious, post-traumatic, or aging/neurodegenerative-related causes; (3) Duration of olfactory dysfunction ≥ 1 month; (4) Willing to receive the treatment and provide written informed consent.

Exclusion Criteria: (1) Nasal lesions, chronic rhinosinusitis with olfactory area obstruction, nasal polyps, severe nasal septum deviation, or a history of nasal surgery; (2) Pregnant or lactating women; (3) Patients with severe cognitive impairment, mental illness, or inability to cooperate with training and follow-up; (4) Patients allergic to herbs ingredients or intranasal corticosteroids; (5) Patients with severe systemic diseases that may significantly affect the evaluation of therapeutic efficacy.

Totally 103 cases were enrolled and 73 cases finished the study (herbs group: 62 enrolled, 26 dropped out, 36 finished; control group: 41 enrolled, 4 dropped out, 37 finished). Based on follow-up records, the main reasons for dropout in the herb group (n = 26) were loss to follow-up (n = 12), poor compliance due to treatment burden (n = 8), subjective feeling of no improvement (n = 4), and mild nasal irritation (n = 2). In the control group (n = 4), reasons were loss to follow-up (n = 2) and poor compliance (n = 2). The etiological distribution was 61.1% post-infectious (22/36), 27.8% aging/neurodegenerative (10/36), and 11.1% post-traumatic (4/36) in the herbs group, and 70.3% post-infectious (26/37), 27.0% aging/neurodegenerative (10/37), and 2.7% post-traumatic (1/37) in the control group, respectively.

Participants in the herbs group received OT using aromatic sachets containing 6 TCM herbs. Training was conducted twice daily, with each session lasting 5-10 min, for a minimum duration of 3 months. After each session, the sachets were promptly stored in sealed bags to preserve volatile integrity. Each set of sachets was used for approximately 1 week, and patients were typically provided with a 1-2 week supply per visit. Participants in the control group received nasal spray of mometasone furoate 200 μg (100 μg per nostril) once daily, for 3 months. Olfactory function was evaluated before and after treatment using the following instruments:

Olfactory Identification Test (OIT): Conducted using the Hanwang Olfactory Function Test Kit, which consists of 16 odorant bottles. Bottles 1- assess odor identification, bottles 9-12 evaluate odor sensitivity, and bottles A-D test odor memory. The test yields a composite score ranging from 0 to 100. Improvement was categorized as follows: an increase of ≥ 15 points indicated “Level 3: significant improvement”, 7-14 points indicated “Level 2: moderate improvement”, and ≤ 6 points indicated “Level 1: slight improvement”.

Olfactory Dysfunction Visual Analog Scale (VAS) [34]: A 10-cm scale where 0 represents complete loss of smell and 10 indicates normal olfactory function. Patients marked the point corresponding to their perceived olfactory ability. A reduction of ≥ 3 points was defined as “Level 3: significant improvement”, a decrease of 1- points as “Level 2: moderate improvement”, and a change of < 1 point as “Level 1: slight improvement”.

Questionnaire of Olfactory Disorders (QOD) [5]: Developed by Hummel and Frasnelli in 2005, this tool quantifies the severity of olfactory loss and its impact on quality of life. Scores range from 0 to 51, with higher scores indicating greater impairment. A reduction of ≥ 7 points was considered “Level 3: significant improvement”, 4-6 points as “Level 2: moderate improvement”, and ≤ 3 points as “Level 1: slight improvement”.

### SPME-GC-MS analysis

Each herbal sample (0.1 g) was precisely weighed into a 20 mL headspace vial, which was then immediately sealed. The analysis was conducted using an Agilent 8890-7250 GC/Q-TOF system equipped with a TriPlus RSH autosampler. Volatile components were extracted using an Agilent DVB/Carbon WR/PDMS solid-phase microextraction fiber (1.10 mm OD, 120 μm) exposed to the headspace at 60 °C for 40 min after a 10 min incubation period. Following extraction, the fiber was thermally desorbed in the injection port at 260 °C for 4 min.

Chromatographic separation was performed on a DB-5MS capillary column (30 m × 0.25 mm × 0.25 μm) with helium carrier gas at a constant flow rate of 0.8 mL/min. The temperature program was set as follows: initial temperature 50 °C held for 3 min, increased to 120 °C at 15 °C/min, then to 205 °C at 3 °C/min, and finally to 260 °C at 10 °C/min with a 7 min hold period. The injection port was maintained at 260 °C with a split ratio of 40:1. Mass spectrometric analysis was conducted in electron impact mode with a mass scan range of 20-450 m/z.

### Identification of odorants from GC-MS analysis

Following GC-MS data acquisition (Fig. S1), raw mass spectra were processed to identify volatile compounds by matching experimental fragmentation patterns against the NIST 2020 mass spectral library. A match factor of ≥ 85 was applied as the threshold for reliable qualitative identification to ensure high spectral similarity between experimental and reference spectra (Table S1-S6).

The relative content of each identified compound was calculated using the peak area normalization method, which served as a semi-quantitative indicator of compound abundance (not representing absolute concentration). To exclude negligible trace constituents, only compounds with a relative peak area percentage > 0.05% were retained. After compiling all qualified compounds from the six TCM herbs and removing duplicate entries of the same constituent shared across different herbs, a total of 72 unique major volatile constituents were defined as the comprehensive odorant profile of the herbal blend (Table S7).

To prioritize compounds for in vitro functional validation, we further narrowed down this list of 72 constituents. First, we retained only compounds with a relative peak area percentage > 0.5% to focus on the most abundant odorants, which were then ranked by their relative content in descending order. From this ranked list, we selected readily available commercial pure standards for dose-response experiments. This filtering process ultimately yielded 16 unique compounds (Table 2), which were subsequently used as ligands for the human OR activation screening and dose-response assays.

### Luciferase assay

The luciferase assay was conducted following an established protocol with the Dual-Glo^™^ Luciferase Assay System (Promega) [35]. Briefly, HEK293T-derived cells were seeded in 96-well plates (Corning). For transfection in each 96-well plate, a mixture containing 1.5 μg of CRE-Luciferase, 0.75 μg of pRL-SV40, 0.75 μg of each human OR, 0.75 μg of RTP1S, and 0.375 μg of M3R was prepared using Lipofectamine2000 transfection reagent (Invitrogen). After 24 h, the transfection medium was replaced with 25 μL of odorant compound diluted in CD293 medium (Gibco), following a rinse with the same solution. Cells were stimulated for 4 h, after which Firefly luciferase (FL) and Renilla luciferase (RL) activities were quantified according to the manufacturer's instructions using a Synergy H1 plate reader (BioTek). The relative luciferase activity was calculated using the formula: (FL/RLsample)/(FL/RLcontrol), where FL/RLsample represents the response of the OR to the test compound, and FL/RLcontrol corresponds to the basal response of the same receptor to the blank control. A total of 390 human ORs were tested.

### Odorant-OR pair experiment screening procedure

The screening workflow consisted of a primary screen followed by two sequential dose-response experiments. For the primary screen, the entire hOR library was stimulated with ten distinct odorant mixtures composed of 20 individual odorants, including 16 odorants derived from TCM herbs and 4 common chemical odorants. Each mixture contained two odorants, applied at a final concentration of 500 µM per odorant. Assay consistency was verified using two positive control pairs: mouse TAAR7f with N,N-dimethylcyclohexylamine and MOR41-1 with coumarin. Negative control wells were transfected with an empty vector, and blank control wells received no odorant. All measurements were performed in triplicate. A receptor was considered potentially activated if the ratio of relative luciferase activity in the experimental group to that in the negative control was ≥ 1.5.

Candidate odorant-receptor pairs identified in the primary screen were subjected to a first dose-response experiment over a concentration range of 5 µM to 3 mM. In this experiment, individual odorants were tested against individual receptors, and each OR was assayed alongside a no-odor control. Each odorant-receptor combination at each concentration was tested in triplicate. Concentration-response data were subjected to curve-fitting analysis. An odorant was classified as a putative agonist if the curve exhibited a clear upward trend with increasing concentration, and the maximum relative luciferase activity ratio between the experimental group and the no-odor control was ≥ 1.5. This experiment also defined the effective concentration range for each responsive odorant-receptor pair.

A second dose-response experiment was then performed using optimized concentration ranges refined from the first assay. All numerical results are reported as mean ± standard deviation (SD) of three independent replicates. Concentration-response data were fitted using a four-parameter logistic model. An odorant was counted as an agonist according to one of the two following sets of criteria, depending on whether a full or semi-quantitative dose-response curve was obtained:

For odorants that yielded a fully quantifiable dose-response curve suitable for nonlinear regression fitting, an odorant was considered an agonist if all of the following were met: (1) the 95% confidence intervals of the fitted top and bottom parameters did not overlap; (2) the standard deviation of the fitted logEC_50_ was less than 1 log unit; (3) the goodness of fit was acceptable with R^2^ ≥ 0.9.

For odorants that produced only a semi-quantitative dose-dependent response (for which a reliable logEC_50_ could not be determined), an odorant was considered an agonist if all of the following were met: (1) the response exhibited a monotonic increase with increasing odorant concentration; (2) the maximum relative response was ≥ 1.5-fold relative to the no-odor control; (3) the goodness of fit was acceptable with R^2^ ≥ 0.9.

### Odorant-OR pair database search

To complement our experimental data, we systematically retrieved known odorant-OR interactions from 2 public databases: M2OR (v2.0) [22] and OlfactionBase (2023 release) [23]. The search was conducted using both chemical names and canonical SMILES strings of the 72 major TCM herb odorants identified by GC-MS and the 4 common chemical odorants. Only entries with a reported response value of 1 (indicating confirmed activation) and involving human ORs were included. The retrieved interactions were then merged with the odorant-OR pairs identified in this study to form a unified dataset. This combined resource comprises 225 TCM odorant-OR pairs (Table S8) and 25 chemical odorant-OR pairs (Table S9), providing a comprehensive basis for comparative analysis of OR activation patterns.

### Homology modeling and docking

Homology models of OR52L1, OR2M3, and OR2T11 were constructed using Swiss-Model [36]. The structure of OR52L1 was modeled based on the cryo-EM structure of consensus OR52 (PDB ID: 8HTI [25]), while OR2M3 and OR2T11 were modeled using the consensus OR2 template (PDB ID: 8UY0 [26]) (Table S10). The quality of all models was assessed via comprehensive stereochemical validation, including Ramachandran plots and MolProbity analysis (Fig. S2, Table S11).

Molecular docking were performed using AutoDock Vina [37]. The ADFR protocol was employed for receptor preprocessing, which included correcting structural issues such as missing loops and steric clashes. Hydrogen atoms were added at physiological pH (7.4), and the protonation states of titratable residues were assigned using the PROPKA module. Receptor structures underwent energy minimization with the OPLS_2005 force field until a convergence Threshold of 0.05 kcal/mol·Å was reached.

The docking grid box for each receptor was centered on the atomic coordinates of the native ligand in its respective template structure (PDB ID: 8HTI for OR52L1; PDB ID: 8UY0 for OR2M3 and OR2T11). A box size of 15 Å was used to encompass the orthosteric binding pocket and adjacent transmembrane residues, with the pocket location independently validated by the DoGSite3 algorithm (Fig. S3). Ligand conformations were prepared and optimized with the Python package Meeko prior to docking and scoring by AutoGrid4 [38]. All docking runs used an exhaustiveness value of 32 to ensure sufficient sampling of ligand binding modes. The resulting poses were ranked by predicted binding affinity (ΔG, kcal/mol), and the top-ranked conformations were visually inspected in PyMOL [39] and Discovery Studio Visualizer to validate their placement within the conserved GPCR binding cavity and to identify key interacting residues.

The reliability of the docking protocol was validated by re-docking experiments using experimental structures: consOR2 (PDB ID: 8UY0), consOR52 (PDB ID: 8HTI), consOR1 (PDB ID: 8UXY), and OR51E2 (PDB ID: 8F76), yielding RMSD values of 1.06 Å, 0.93 Å, 0.83 Å, and 1.25 Å between crystal and docked ligand poses, respectively (Fig. S4).

### Molecular dynamic simulations

MD simulations were performed using GROMACS 2020 [40] with the ff14SB force field [41], starting from the docked complex structure. The system was prepared using the pdb4amber tool from AMBERTOOLS 2023 [42] and solvated in a TIP3P water box. Protein heavy atoms were subjected to a three-stage energy minimization protocol with progressively reduced positional restraints (5, 3, and 0 kcal/(mol·Å^2^)). The system was then gradually heated from 0 to 300 K over 1 ns in the NVT ensemble using the Nosé-Hoover thermostat [43], followed by a 10-ns NPT equilibration phase to stabilize the solvent environment. Positional restraints of 3 kcal/(mol·Å^2^) were maintained during both heating and equilibration stages. Finally, three independent 300‑ns production simulations were carried out under NPT conditions without any positional restraints.

### Free energy calculation

The binding free energies of ligand-receptor complexes were estimated using the Molecular Mechanics Generalized-Boltzmann Surface Area (MM/GBSA) [44] method from AMBERTOOLS 2023 [42], calculated from the final 100 ns of each independent MD trajectory.

### Statistics

All statistical analyses for clinical data were conducted using SPSS 26.0 and G*Power 3.1.9.7. Quantitative data are expressed as mean ± SD. Within-group comparisons (pre- vs. post-treatment) were performed using paired samples t-tests. Between-group comparisons of improvement scores were performed using independent samples t-tests. To control for multiple comparisons across the three outcome measures (OIT, VAS, QOD), the Bonferroni correction was applied, with the significance level set at α = 0.017 (0.05/3) for each outcome. Cohen’s d was calculated for between-group effect sizes. Post-hoc power analysis was performed based on observed effect sizes using G*Power 3.1.9.7. A p < 0.05 was considered statistically significant for uncorrected comparisons, and p < 0.017 for Bonferroni-corrected comparisons.

For the in vitro OR activation assays, dose–response curves were generated from three independent experiments (N = 3), and the derived parameters (Emax, logEC_50_) are reported as mean ± SD. Data analysis was conducted using GraphPad Prism 8.2.1.

## Results

### TCM herbs improve olfactory function

**Fig. 1:**
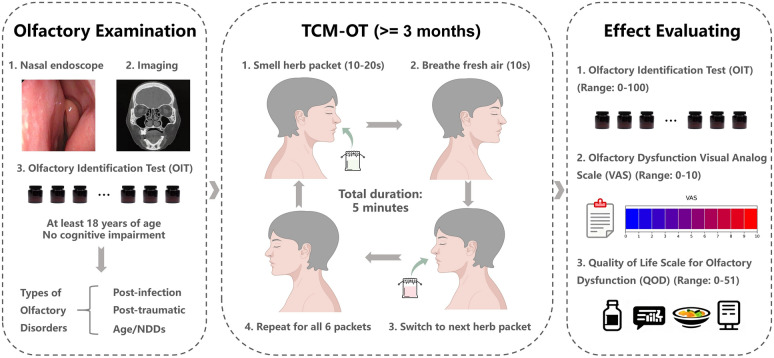
Flowchart of the TCM-OT study. The diagram outlines patient recruitment, the TCM-OT protocol involving sequential exposure to six different TCM herb packets, and the assessment of therapeutic effect using quantification tables (OIT, VAS, QOD)

A total of 73 patients with OD successfully completed the treatment program (36 in herb group, 37 in control group). The herb group used aromatic extracts from six TCM herbs: Borneol, Caryophylli Flos, Dried Ginger, Pogostemon Cablin, Myristica Fragrans, and Magnoliae Flos for 12 weeks. The control group used mometasone furoate nasal spray for 12 weeks. All participants were initially diagnosed via nasal endoscopy, imaging, and OIT, with etiologies categorized as post-infection, post-traumatic, or age-related/neurodegenerative. The TCM-OT treatment plan involved sequential inhalation of each herbal packet for 10-20 s, interspersed with breathing fresh air, totaling around 5 min per session (Fig. 1), performed twice daily. The OT treatment plan for the control group consisted of one spray per nostril, once daily.

Following intervention, significant improvements were observed in both groups (Table 1). In the herb group (n = 36), OIT scores increased from 42.06 ± 14.40 to 53.36 ± 12.96 (p < 0.001), VAS scores decreased from 5.83 ± 1.34 to 4.47 ± 1.13 (p < 0.001), and QOD scores decreased from 23.22 ± 8.29 to 19.19 ± 6.80 (p < 0.001). In the control group (n = 37), OIT scores increased from 45.11 ± 7.48 to 51.32 ± 9.00 (p < 0.001), VAS scores decreased from 5.92 ± 0.89 to 4.95 ± 1.15 (p < 0.001), and QOD scores decreased from 22.92 ± 3.09 to 19.86 ± 3.43 (p < 0.001).

**Table 1:** Olfactory function scores before and after treatment. Statistical significance was determined by paired t-test

Olfactory scoring method	Before treatment	After treatment	Change (after - before)	t value	*P* value	
Olfactory Identification Test (OIT)					Within group	Between group
Herb (n = 36)	42.06 ± 14.40	53.36 ± 12.96	+ 11.31 ± 6.30	10.77	< 0.001	< 0.001^*^
Control (n = 37)	45.11 ± 7.48	51.32 ± 9.00	+ 6.22 ± 4.99	7.58	< 0.001	
Olfactory Dysfunction Visual Analog Scale (VAS)						
Herb (n = 36)	5.83 ± 1.34	4.47 ± 1.13	− 1.36 ± 0.93	− 8.78	< 0.001	0.030
Control (n = 37)	5.92 ± 0.89	4.95 ± 1.15	− 0.97 ± 0.64	− 9.18	< 0.001	
Questionnaire of Olfactory Disorders (QOD)						
Herb (n = 36)	23.22 ± 8.29	19.19 ± 6.80	− 4.03 ± 2.66	− 9.10	< 0.001	0.057
Control (n = 37)	22.92 ± 3.09	19.86 ± 3.43	− 3.05 ± 1.45	− 12.79	< 0.001	

Between-group comparisons showed that the improvement in OIT was significantly greater in the herb group compared to the control group (11.31 ± 6.30 vs. 6.22 ± 4.99, p < 0.001, Cohen’s d = 1.150). Improvements in VAS and QOD showed trends favoring the herb group (VAS: − 1.36 ± 0.93 vs. − 0.92 ± 0.76; QOD: − 4.03 ± 2.66 vs. − 3.05 ± 1.45) but did not reach statistical significance after Bonferroni correction (p > 0.017).

To further evaluate therapeutic efficacy, patients were stratified into 3 improvement levels (see method) for each metric in both groups.

**Fig. 2:**
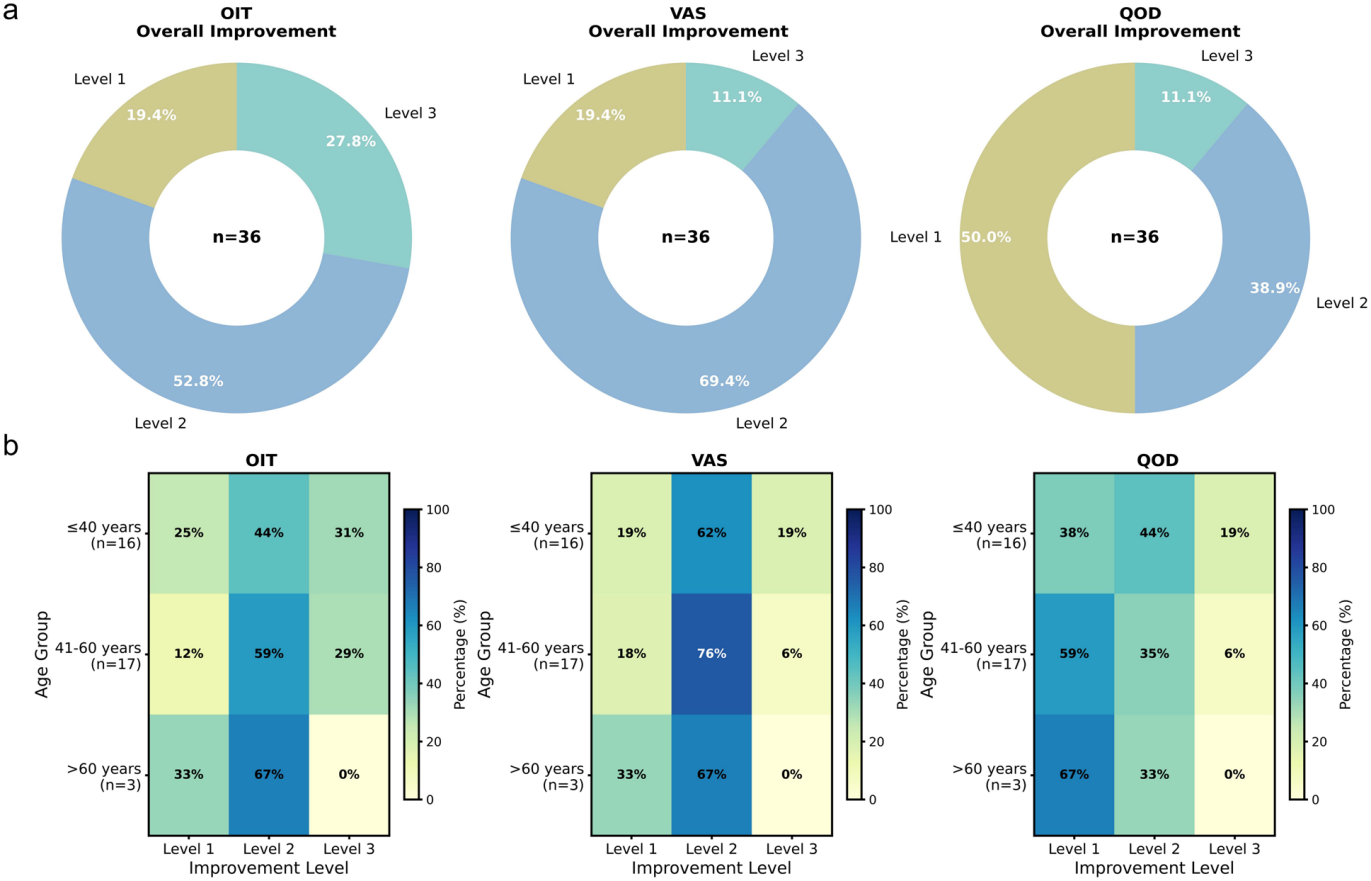
Efficacy assessment of TCM-OT. **a** Overall improvement: Pie charts showing the proportion of patients achieving different levels of improvement (Level 1: slight; Level 2: moderate; Level 3: significant) for each outcome measure (OIT, VAS and QOD) across all patients. **b** Improvement by age: Heatmaps showing the distribution of improvement levels for OIT, VAS and QOD scores among patients of different ages following the therapeutic intervention

Herb group (n = 36): The OIT scores showed the highest proportion of significant improvement (Level 3: 27.8%, 10/36), followed by moderate improvement (Level 2: 52.8%, 19/36). The VAS and QOD scores showed more modest rates of significant improvement (Level 3: 11.1% each, 4/36). Nearly half of the patients exhibited slight improvement (Level 1: 50.0%, 18/36) in QOD, suggesting that odor identification improved more readily than perceived dysfunction and quality-of-life impacts (Fig. 2a).

Control group (n = 37): The OIT scores showed a moderate rate of significant improvement (Level 3: 16.2%, 6/37), while no patients achieved significant improvement in VAS (Level 3: 0%) or QOD (Level 3: 0%). Most patients showed slight improvement in OIT (Level 1: 81.1%, 30/37) (Fig. S5a).

Comparison: The herb group showed higher rates of significant improvement compared to the control group for all metrics, particularly for OIT (27.8% vs. 16.2%) (Figs. 2a and S5a).

Age-stratified analysis revealed that in the herb group, no patients over 60 (not include 60) years attained significant improvement (Level 3) in OIT (n = 3, mean improvement 8.33 ± 3.51). In the control group, one patient over 60 years achieved significant improvement (n = 4, 1/4 with Level 3 improvement) (Figs. 2b and S5b). Subgroup analysis by etiology showed that TCM-OT consistently improved OIT scores across all three etiological categories, with the most pronounced numerical benefit observed in post-traumatic cases, though the small sample size in this subgroup precludes firm conclusions (Table S12). No clear correlations were observed with sex or etiology, though the limited sample size (36 in herb group, 37 in control group) precludes definitive conclusions (Fig. S6-S7).

The herb group maintained a trend of greater improvement compared to the control group across all metrics, particularly for the primary outcome (OIT). However, larger randomized trials are needed to confirm these observations.

Comparison with the standard OT protocol by Hummel et al. [16] showed that the 27.8% significant improvement rate in our herb group is numerically comparable to the approximately 30% rate reported with conventional four odorant training. However, this indirect comparison is limited by differences in patient populations, outcome measures, and study designs.

These findings collectively demonstrate that TCM-OT elicits statistically and clinically meaningful improvements in olfactory function. The differential response across outcome measures underscores the olfactory recovery, where objective identification may improve more readily than patient-reported burden. The age-dependent effect highlights the potential influence of physiological aging on regenerative capacity, aligning with previous evidence of diminished olfactory neurogenesis in older adults. While the small cohort limits generalizability, the absence of sex- or etiology-based differential outcomes suggests that herb odorants may offer promising a broad-spectrum approach amenable to diverse patient subgroups. Further studies with larger samples and longer follow-up are warranted to validate these observations and optimize personalized treatment protocols.

### TCM herb odorants exhibit diverse physicochemical properties

**Fig. 3:**
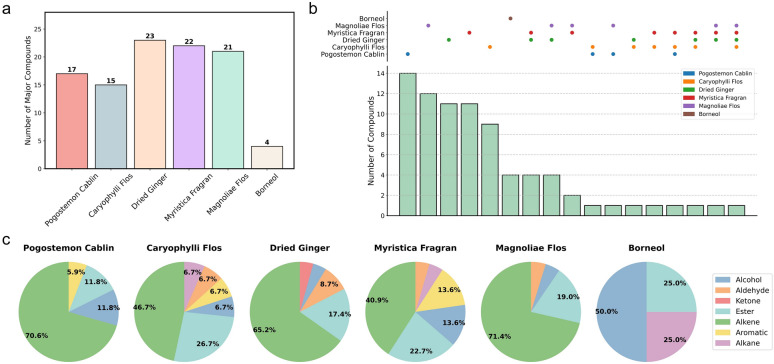
Phytochemical composition of the 6 TCM herbs analyzed by GC-MS. **a** The number of major chemical constituents (relative content ≥ 0.05%) identified in each TCM. **b** A bar plot illustrating the overlap of major constituents among the six herbs and highlighting the numbers of shared and unique compounds. **c** The proportional distribution of different chemical classes (alcohols, aldehydes, ketones, esters, alkenes, aromatics, and others) among the identified major constituents

Having demonstrated the clinical efficacy of TCM-OT compared with corticosteroid treatment (control group), we next investigated the chemical complexity of TCM herb odorants relative to the four classic chemical odorants used in conventional olfactory training. We characterised their volatile compositions using GC-MS analysis. A total of 72 major constituents (match factor ≥ 85 and relative content ≥ 0.05%) were identified across the 6 herbs (Table S7), with Dried Ginger and Myristica Fragran containing the highest number of major compounds (23 and 22, respectively), while Borneol contained only 4 (Fig. 3a). Analysis of compound overlap revealed that the majority of constituents were herb-specific, with 4 out of 6 herbs possessing over 10 unique compounds (Fig. 3b). Only a limited number of components were shared among 3 or more herbs, including butyl isobutyl phthalate, isovanillin, 3-carene, and $\gamma$-cadinene. Chemical class distribution analysis demonstrated that alkenes constituted the most abundant group in 5 of the 6 herbs. Esters and alcohols were also widely present (Fig. 3c).

**Fig. 4:**
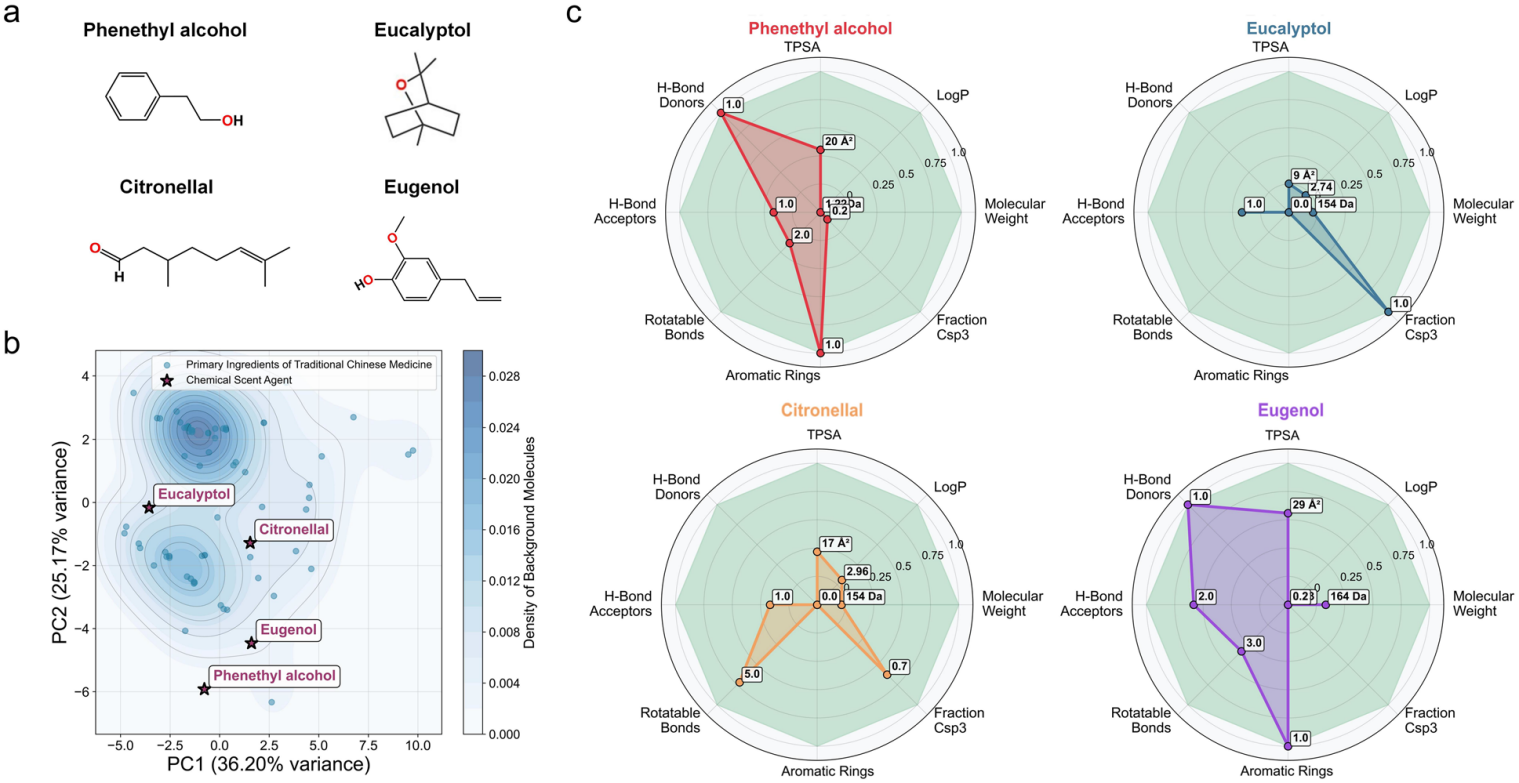
Comparative analysis of major 72 odorants identified in the six TCM herbs and 4 common chemical odorants. **a** Molecular structures of chemical odorants. **b** PCA plot of the odor space, depicting the distribution of major herb odorants (blue dots) and the chemical odorants (red stars). **c** Radar chart comparing the chemical odorants against the background profile of the major herb odorants (green area) across 8 key physicochemical property dimensions

To compare our TCM-based approach with previously established OT methods using defined chemical odorants, we analyzed 4 commonly used scent molecules: phenethyl alcohol (rose-like), eucalyptol (eucalyptus), citronellal (citrus), and eugenol (clove) (Fig. 4a) [16]. Principal Component Analysis (PCA) revealed that the herb odorants occupied a broad physicochemical space forming 2 distinct clusters, whereas the 4 chemical odorants were situated within this expansive TCM region though not at the cluster centers (Fig. 4b). Radar chart analysis revealed that the molecular properties of all 4 chemical odorants were fully encompassed by the distribution of the herb odorants across 8 key descriptors, with the majority of values centrally clustered within this range and only a few near its periphery (Fig. 4c). While the chemical odorants exhibited distinct and complementary profiles that rationalize their combination in previous studies, the herbs collectively provided a much broader spectrum of chemical diversity.

Together, this comparative analysis demonstrate that while selected chemical odorants represent a reasonable combination for OT, the herbs offer substantially greater chemical complexity and diversity. This comprehensive phytochemical profile likely provides a more robust stimulus for OR activation.

### Broad and novel activation of human ORs by TCM herb odorants

**Fig. 5:**
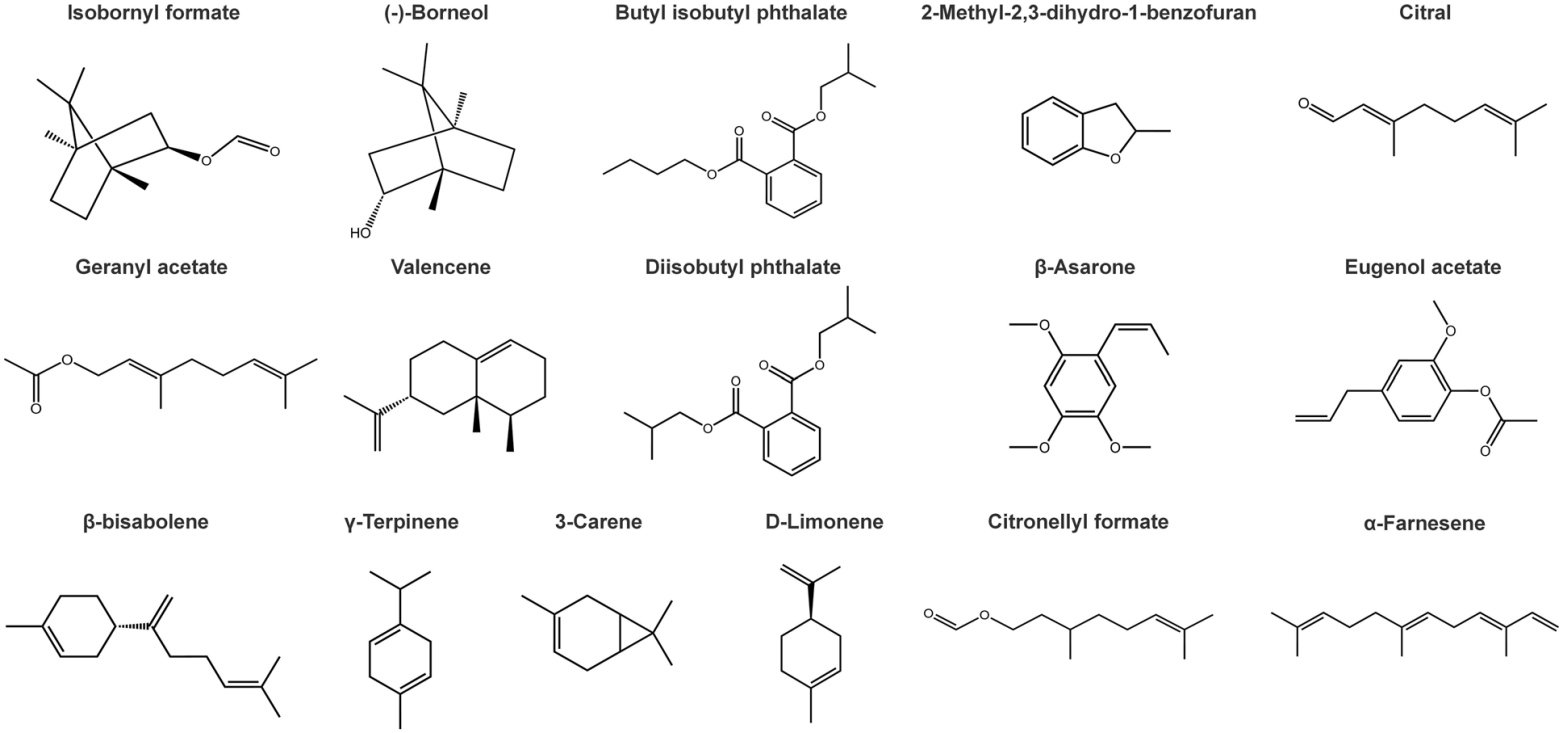
Molecular structures of 16 TCM herb odorants

To functionally characterize the interactions between TCM herb odorants and human ORs, we first selected 16 major volatile constituents (Table 2, Fig. 5) from the 6 herbs based on their high relative content and commercial availability. These compounds were initially screened against a library of approximately 400 human ORs using a luciferase reporter assay, followed by comprehensive dose-response validation. Of the 16 herb odorants tested, 10 activated at least one OR, resulting in 34 well-defined odorant-OR interaction pairs (Table 3, Fig. S8). Among the herb odorants, Eugenol acetate emerged as the most promiscuous ligand, activating 11 distinct ORs, followed by (-)-Borneol and Diisobutyl phthalate, which activated 7 and 6 ORs, respectively. The strongest responses were observed for Eugenol acetate in combination with OR2T6 and OR10G7, both exhibiting high Emax values (23.92 ± 1.94 and 25.77 ± 3.85, respectively) and potent logEC_50_ values below − 4.7, indicating high efficacy and potency. Several other pairs also showed notable activation, including (-)-Borneol with OR2T11 (Emax = 29.27 ± 2.95) and Diisobutyl phthalate with OR12D2 (Emax = 26.74 ± 1.89). Notably, among the 34 functional pairs identified for herb odorants, 23 were novel interactions not previously reported (Table 3, Table S8), including 4 newly deorphaned receptors: OR14I1, OR2T6, OR5H15, and OR2F2. This highlights the rich and unexplored bioactivity of TCM herb odorants.

**Table 2:** Candidate odorants selected from six TCM herbs by GC-MS for functional analysis of human ORs

TCM	Odorant	CAS	Chemical formula	Peak area percentage (%)
Borneol	Isobornyl formate	1200-67-5	C11H18O2	6.44
	(-)-Borneol	464-45-9	C10H18O	2.55
Caryophylli Flos	Butyl isobutyl phthalate	17851-53-5	C16H22O4	3.73
	2-Methyl-2,3-dihydro-1-benzofuran	1746-11-8	C9H10O	3.22
Dried Ginger	Citral	5392-40-5	C10H16O	5.30
	Geranyl acetate	105-87-3	C12H20O2	2.53
	γ-Terpinene	99-85-4	C10H16	1.45
	3-Carene	13466-78-9	C10H16	0.66
Pogostemon Cablin	Valencene	4630-07-3	C15H24	7.17
	Diisobutyl phthalate	84-69-5	C16H22O4	1.43
	Butyl isobutyl phthalate	17851-53-5	C16H22O4	0.81
Myristica Fragran	β-Asarone	5273-86-9	C12H16O3	4.01
	Eugenol acetate	93-28-7	C12H14O3	2.63
	γ-Terpinene	99-85-4	C10H16	1.67
	β-bisabolene	495-61-4	C15H24	0.77
	Butyl isobutyl phthalate	17851-53-5	C16H22O4	0.77
Magnoliae Flos	3-Carene	13466-78-9	C10H16	5.12
	D-Limonene	5989-27-5	C10H16	4.18
	Citronellyl formate	105-85-1	C11H20O2	2.76
	α-Farnesene	502-61-4	C15H24	1.89

After removing duplicates, 16 unique compounds remain

**Table 3:** Activation of human ORs by TCM herb odorants

Odorant	OR	Emax	LogEC_50_
Isobornyl formate	OR2W1	N/A	− 2.15 ± 0.15
(-)-Borneol	OR2W1	9.71 ± 0.47	− 2.54 ± 0.04
(-)-Borneol	OR2C1	N/A	N/A
(-)-Borneol	OR2M3	10.85 ± 0.92	− 3.12 ± 0.07
(-)-Borneol	OR2T11	29.27 ± 2.95	− 2.35 ± 0.04
(-)-Borneol	OR10X1	10.94 ± 0.59	− 2.60 ± 0.06
(-)-Borneol	OR52L1	7.65 ± 0.70	− 2.48 ± 0.04
(-)-Borneol	OR7A17	7.21 ± 0.58	− 2.46 ± 0.03
Butyl isobutyl phthalate	OR9I1	9.55 ± 0.71	− 2.82 ± 0.07
2-Methyl-2,3-dihydro-1-benzofuran	OR5K1	3.22 ± 0.41	− 4.00 ± 0.24
Citral	OR1A1	N/A	N/A
Citral	OR3A3	12.26 ± 1.66	− 2.85 ± 0.06
Citral	OR7E24	N/A	N/A
Geranyl acetate	OR1A1	N/A	N/A
Geranyl acetate	OR2W1	3.15 ± 0.59	− 3.06 ± 0.31
Diisobutyl phthalate	OR14A2	6.33 ± 0.27	− 2.81 ± 0.05
Diisobutyl phthalate	OR14I1	8.44 ± 1.06	− 2.70 ± 0.07
Diisobutyl phthalate	OR12D2	26.74 ± 1.89	− 2.47 ± 0.05
Diisobutyl phthalate	OR5T2	N/A	− 2.69 ± 0.16
Diisobutyl phthalate	OR3A3	6.94 ± 0.36	− 2.63 ± 0.04
Diisobutyl phthalate	OR4D2	N/A	− 2.46 ± 0.15
Eugenol acetate	OR2T6	23.92 ± 1.94	− 4.87 ± 0.09
Eugenol acetate	OR5H15	2.13 ± 0.18	− 2.52 ± 0.09
Eugenol acetate	OR2J3	6.01 ± 0.93	− 3.55 ± 0.15
Eugenol acetate	OR2F2	2.44 ± 0.16	− 2.64 ± 0.08
Eugenol acetate	OR51A7	2.41 ± 0.13	− 2.65 ± 0.07
Eugenol acetate	OR6T1	N/A	− 2.55 ± 0.07
Eugenol acetate	OR10G4	N/A	N/A
Eugenol acetate	OR10G7	25.77 ± 3.85	− 4.77 ± 0.21
Eugenol acetate	OR2W1	N/A	N/A
Eugenol acetate	OR10A5	N/A	N/A
Eugenol acetate	OR52A4	N/A	N/A
D-Limonene	OR1A1	N/A	N/A
Citronellyl formate	OR2J3	1.95 ± 0.16	− 2.74 ± 0.16

“N/A” indicates a detectable response for which specific efficacy or potency parameters could not be fitted

For direct comparison of the OR activation profiles between TCM odorants and conventional chemical odorants, we performed parallel dose-response screening of the 4 canonical odorants used in standard OT protocols (eugenol, eucalyptol, citronellal, and phenethyl alcohol) against the same human OR library. This assay identified 11 odorant-OR interaction pairs, 10 of which were novel pairings (Table 4, Fig. S9, Table S9). Among the four odorants, eugenol exhibited the broadest activation capacity, targeting 6 distinct ORs. The most potent activation was observed for eugenol with OR2T6 (Emax = 20.77 ± 0.87). Similar to the pattern seen with herb odorants, OR1 and OR2 families were also activated by multiple odorants.

**Table 4:** Activation of human olfactory receptors by 4 chemical odorants

Odorant	CAS	OR	Emax	LogEC_50_
Eugenol	97-53-0	OR2J3	4.98 ± 0.32	− 3.49 ± 0.08
		OR10G4	5.03 ± 0.28	− 3.59 ± 0.08
		OR1A1	6.77 ± 0.31	− 3.19 ± 0.05
		OR2C1	N/A	− 2.72 ± 0.27
		OR2T6	20.77 ± 0.87	− 5.21 ± 0.07
		OR10G7	N/A	N/A
Citronellal	106-23-0	OR2J3	3.50 ± 0.26	− 3.00 ± 0.08
		OR1A1	N/A	− 2.88 ± 0.21
		OR5K1	2.91 ± 0.48	− 2.88 ± 0.19
Phenylethyl alcohol	60-12-8	OR2J3	10.15 ± 1.04	− 3.17 ± 0.10
		OR11H4	9.74 ± 1.73	− 3.27 ± 0.21
Eucalyptol	470-82-6	–	–	–

“-” indicates that no activating odorant-OR pair was detected for this odorant in our experiments. “N/A” indicates a detectable response for which specific efficacy or potency parameters could not be fitted

**Fig. 6:**
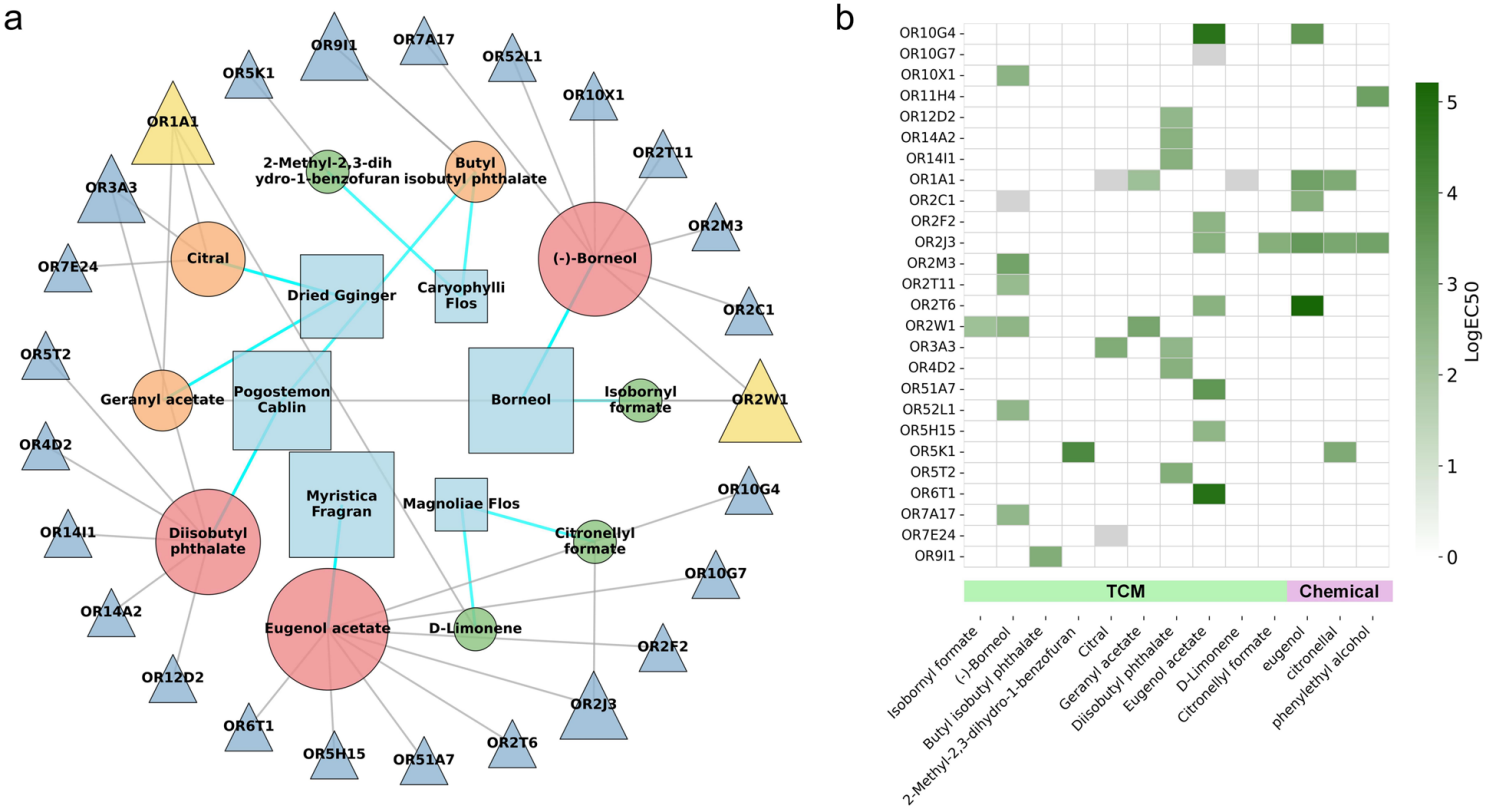
OR activation profiles of TCM odorants and chemical odorants. **a** Network of TCM odorant-OR activation interactions, where squares represent TCMs, circles represent odorants, and triangles represent ORs. **b** Heatmap of the absolute values of logEC_50_ for OR activation by TCM odorants and chemical odorants. Darker colors indicate stronger responses. Grey cells indicate activation was detected, but the logEC_50_ could not be accurately fitted

The network diagram (Fig. 6a) and the logEC_50_ heatmap (Fig. 6b) collectively illustrate the activation profiles of TCM odorants and conventional chemical odorants. TCM odorants activated a substantially larger number of human ORs than the four chemical odorants. Notably, all ORs activated by the chemical odorants — with the sole exception of OR11H4 — were also activated by TCM odorants, indicating that the herbal volatiles largely encompass the receptor targets of standard OT odorants while providing a much broader activation spectrum. These results demonstrate that TCM herb odorants engage a far more diverse and expansive set of ORs compared to conventional chemical odorants, offering a molecular basis for the enhanced therapeutic efficacy of TCM-OT and supporting its potential to deliver comprehensive, multi-pathway stimulation to the olfactory system.

### TCM herb odorants activate all 17 human OR families

We performed a comprehensive comparison of the OR activation patterns between TCM herb odorants and common chemical odorants by integrating our experimental activation pairs with publicly available datasets from databases (M2OR [22] and OlfactionBase [23]). These databases collect interactions measured by diverse bioassay platforms, cell expression systems, and experimental conditions, which broaden the coverage and enhance the reliability of cross-comparison.

While our GC-MS analysis identified 72 major odorants in the 6 herbs, the publicly available databases contained OR activation information for only 7 of these 72 molecules. By combining our experimentally validated pairs (34 pairs) with those from the databases, we assembled a unified dataset of 225 odorant-OR pairs for the herb odorants (Table S8). For the 4 common chemical odorants (phenethyl alcohol, eucalyptol, citronellal, eugenol), our parallel functional screening identified 11 odorant-OR interaction pairs. After incorporating data from public databases, the total number of activation pairs increased to 25 (Table S9). This integrated approach enabled a systematic comparison of OR family activation profiles between the two groups.

**Fig. 7:**
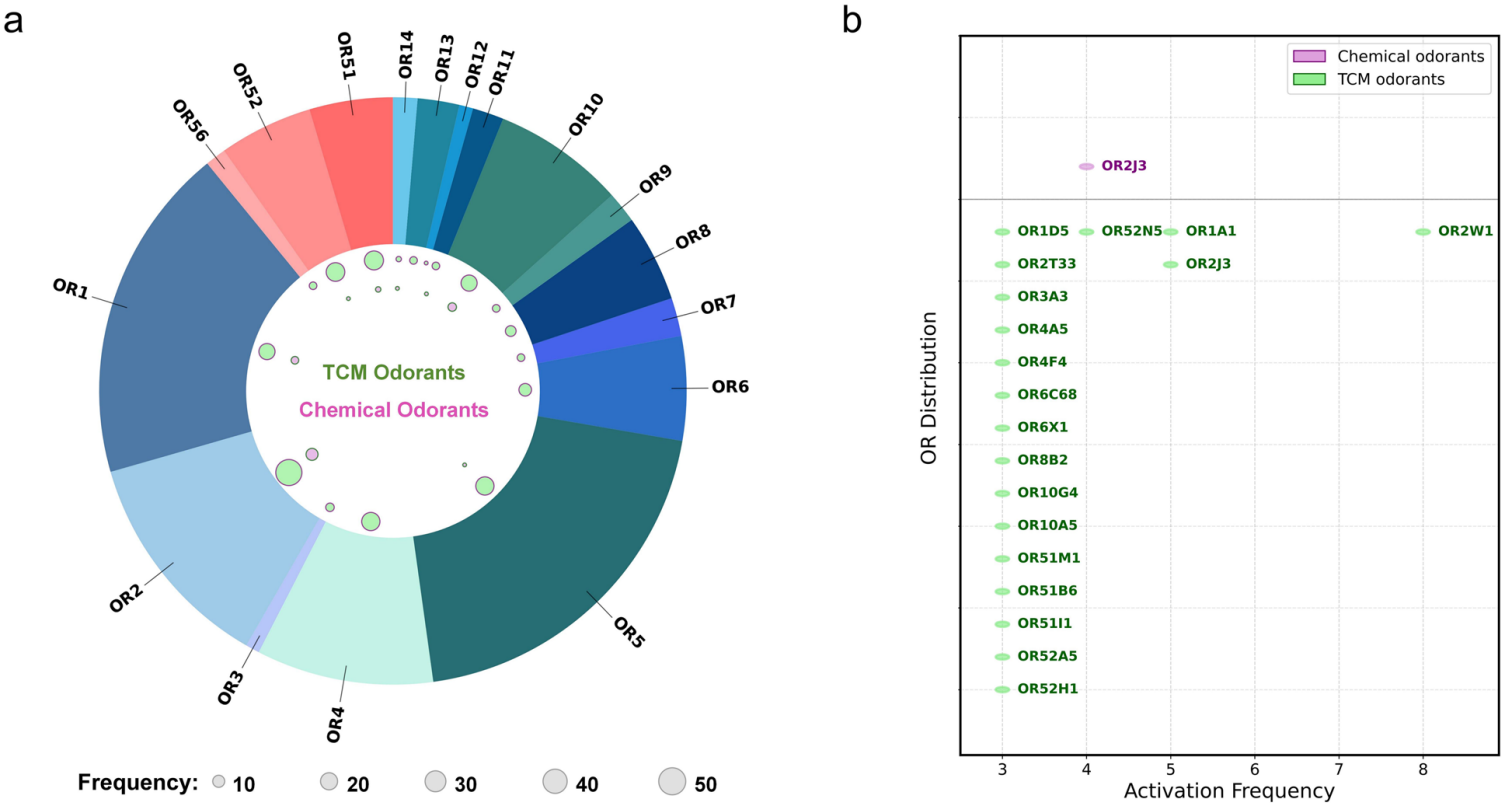
Analysis of OR activation profiles. **a** The number of ORs activated by odorants. Green circles represent major TCM ingredients, and pink circles represent the four chemical odorants. Activation data are derived from the union of this study and publicly available databases. **b** The subset of ORs that were activated by more than two odorants

Analysis of the OR families activated revealed striking differences in the breadth of OR engagement. The TCM herb odorants activated receptors across all 17 families of human ORs (Class I: OR51, OR52, OR56; Class II: OR1-14). The most frequently activated families were OR2, OR51, and OR52 (Fig. 7a). By comparison, the common chemical odorants activated ORs from only 8 out of the 17 families (OR1, OR2, OR5, OR10, OR11, OR14, OR51, OR52) (Fig. 7a). This pattern holds true whether considering only our experimental activation pairs or the combined dataset including database entries: TCM odorants consistently engage a substantially broader range of OR families than the chemical odorants. We acknowledge that the OR activation data, especially those retrieved from public databases, may be subject to limitations such as incomplete coverage of all possible odorant-OR pairs and potential platform-dependent variability. Nevertheless, the consistent and marked difference in family-wide activation indicates that the complex volatile mixture of TCM herbs provides a much wider spectrum of OR engagement compared to the limited set of chemical odorants.

We further identified ORs that were activated by multiple odorants (Fig. 7b). Among the TCM herb odorants, 19 ORs were activated by three or more different ligands. The most promiscuous receptor was OR2W1, which responded to 8 distinct herb odorants, confirming its broad tuning properties. OR1A1 and OR2J3 were also highly responsive, each being activated by 5 different odorants. Meanwhile, among the chemical odorants, OR2J3 was the only receptor activated by all 4 odorants.

This observation supports the hypothesis that the therapeutic efficacy of TCM-OT may involve two complementary mechanisms: broad activation across all 17 OR families, coupled with particularly strong stimulation of broadly-tuned ORs. The comprehensive activation of all OR families by herb odorants, compared with the narrower receptor engagement of common chemical odorants, suggests a potentially more robust and holistic stimulation of the olfactory system. Such broad activation could, in theory, be beneficial for promoting neuroregeneration and functional recovery in the olfactory epithelium, although this remains a hypothesis. Furthermore, the repeated and potent stimulation of promiscuous receptors (e.g., OR2W1, OR1A1, and OR2J3) by herb odorants might play a role in maintaining receptor sensitivity and supporting the survival and maturation of olfactory sensory neurons. We acknowledge that these mechanistic links between OR activation patterns and clinical outcomes are currently speculative. Future studies are needed to test this hypothesis by investigating the precise signaling pathways activated by these herb odorants and their direct effects on olfactory neurogenesis in relevant models of OD.

### (-)-Borneol engages diverse ORs through conserved and receptor-adaptive mechanisms

**Fig. 8:**
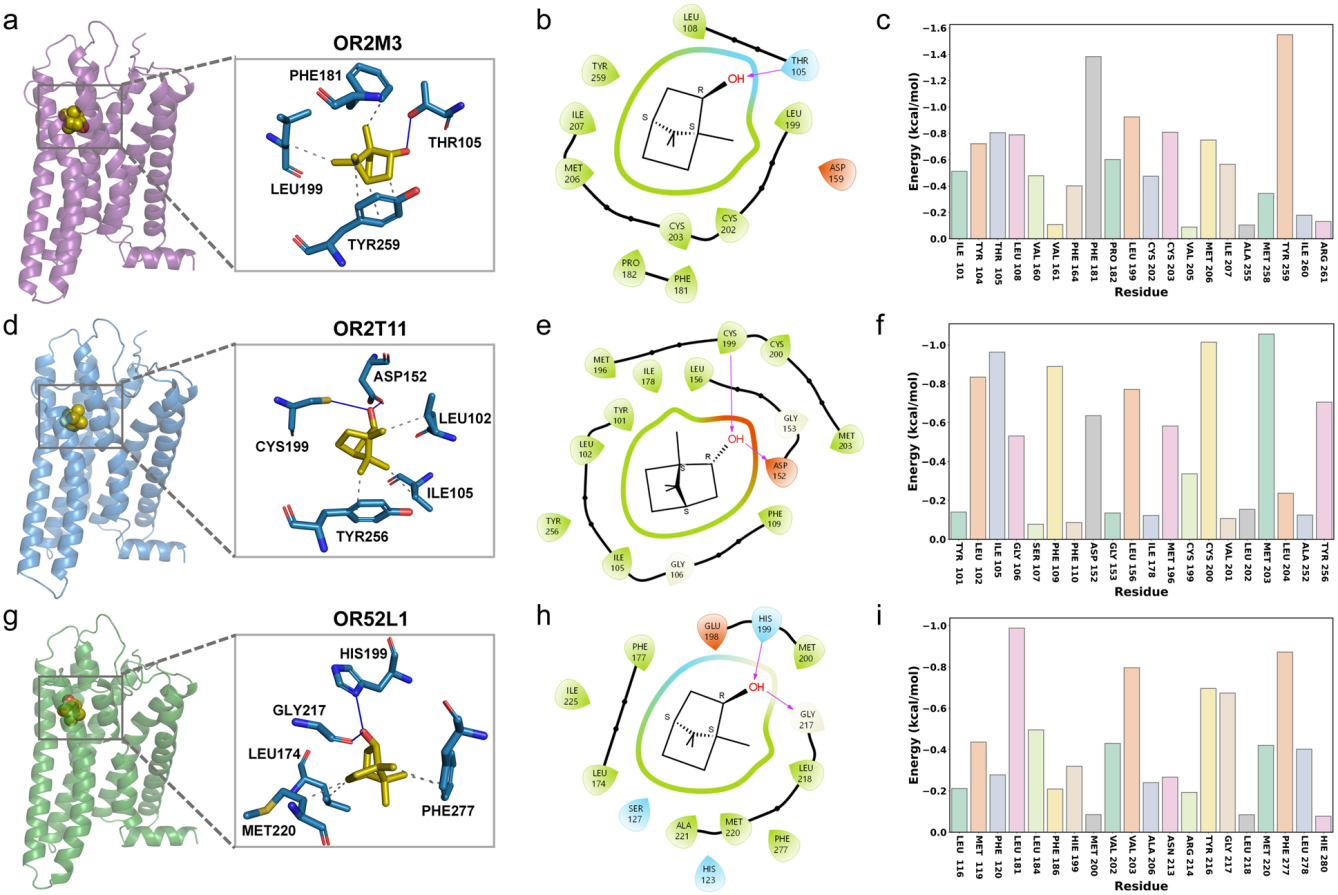
Molecular interaction and binding analysis of (-)-Borneol with three ORs. **a**, **d**, **g** Three-dimensional binding poses of (-)-Borneol within the binding pockets of OR2M3, OR2T11, and OR52L1, respectively. **b**, **e**, **h** Two-dimensional diagrams illustrating detailed ligand-receptor interactions within the 4 Å binding pocket surrounding (-)-Borneol for OR2M3, OR2T11, and OR52L1, respectively. **c**, **f**, **i** Molecular Mechanics Poisson-Boltzmann Surface Area (MM/GBSA) binding free energy decomposition for the top 15 contributing residues from MD simulations of (-)-Borneol bound to OR2M3, OR2T11, and OR52L1, respectively

Our functional screening identified (-)-Borneol as a multi-target ligand capable of activating 7 human ORs (OR2W1, OR2C1, OR2M3, OR2T11, OR10X1, OR52L1, and OR7A17), spanning both Class I and Class II OR subfamilies. To elucidate the structural basis of this broad recognition, we first performed molecular docking for all 7 responsive receptors to characterize their binding modes (Fig. 8, Fig. S10, Table S13). We then selected 3 representative receptors for further MD simulations to validate binding stability and energetic properties: OR2M3 and OR2T11 (Class II), and OR52L1 (Class I). All MD simulations were performed in triplicate independent runs, and the RMSD profiles of the systems confirmed that the simulations reached stable equilibration (Fig. S11).

OR2M3 has been characterized as a highly specific receptor for the onion key food odorant 3-mercapto-2-methylpentan-1-ol, highlighting its role in food-related odor perception [45]. OR2T11 represents an intriguing case as a segregating pseudogene, with certain alleles encoding potentially functional receptors, contributing to population variation in odor perception [46]. Recent genomic and transcriptomic studies have associated OR52L1 with several neurological and sensory disorders [47, 48]. These findings collectively suggest that the multi-receptor targeting capability of (-)-Borneol may engage not only canonical olfactory pathways but also intersect with broader physiological processes, warranting further investigation into its potential therapeutic applications in neurological disorders.

Molecular docking revealed that (-)-Borneol binds stably within the canonical orthosteric pocket of all three receptors (Fig. 8a, d, g). A conserved recognition pattern emerged: the hydroxyl group of (-)-Borneol forms a pivotal hydrogen bond with a polar residue in the binding pocket (Thr105^3×33^ in OR2M3, Asp152^4×56^/Cys199^5×43^ in OR2T11, Ser127^3×37^/His199 in OR52L1), while its bicyclic terpenoid scaffold engages in extensive hydrophobic contacts with surrounding residues (Fig. 8b, e, h). This binding mode is also consistent with the docking results of (-)-Borneol with the other 4 ORs (Table S13).

MM/GBSA binding free energy calculations and decomposition analysis were performed based on the MD trajectories (Table S14). OR2M3 exhibited the lowest binding free energy among the three, which was consistent with our experimental observation that OR2M3 had the lowest logEC_50_ value (i.e., the highest activation potency) for (-)-Borneol, validating the reliability of our computational predictions. Further free energy decomposition highlighted key stabilizing residues for each system, such as Phe181/Tyr259^6×55^ in OR2M3, Ile105^3×36^/Cys200^5×43^/Met203^5×46^ in OR2T11, and Leu181^4×64^/Phe277^6×55^ in OR52L1, which collectively anchor the ligand through van der Waals and hydrophobic interactions (Fig. 8c, f, i). Although the overall binding mode remains unchanged, (-)-Borneol can adapt its structure to the unique binding landscape of each receptor. This structural plasticity, enabled by its moderately rigid yet compact molecular structure, allows (-)-Borneol to maintain essential polar anchoring while optimising hydrophobic complementarity across divergent ORs.

In summary, our integrated computational approach reveals both conserved and receptor-specific interaction patterns underlying the multi-target recognition of (-)-Borneol. The compound engages predominantly with conserved transmembrane residues through a combination of hydrogen bonding and hydrophobic interactions, yet adapts to each receptor's unique binding landscape. This structural plasticity may explain its ability to activate multiple ORs across different phylogenetic classes, providing molecular insights into the broad reactivity of this natural terpenoid and its potential applications in OD therapy through multi-receptor targeting.

## Discussion

Our study provides a systematic integration of clinical observations and molecular investigations to elucidate the efficacy and mechanisms of TCM herbs in treating OD. Clinically, the preliminary results indicate that the herb group showed greater improvement than the control group, particularly for the primary outcome (OIT). TCM-OT significantly improved OIT scores (p < 0.001 after Bonferroni correction), with trends favoring TCM-OT for VAS and QOD that did not reach statistical significance after multiple comparison correction. These outcomes suggest potential advantages over conventional nasal spray treatment, though the non-randomized design limit direct comparability. Notably, efficacy appeared reduced in elderly patients (≥ 60 years), with no patients in the herb group achieving significant improvement (Level 3) in OIT, underscoring the potential need for personalized protocols in this population. However, these observations require confirmation in larger randomized controlled trials [49, 50].

At the molecular level, TCM herb odorants exhibited substantially greater physicochemical diversity than common chemical odorants, as evidenced by broader distribution in PCA and radar chart analyses. In our experimental screening of 16 major herb odorants, TCM odorants activated a larger number of ORs and OR families than the 4 chemical odorants. By integrating our experimentally validated pairs (including 33 novel ones) with public database entries, the combined dataset suggested that TCM herb odorants collectively activate all 17 human OR families — a striking contrast to the limited family engagement of conventional chemical odorants. This observation leads to the hypothesis that the superior receptor coverage of TCM-OT may contribute to its therapeutic efficacy, although this remains to be directly tested. Structural analysis further revealed a potential multi-receptor binding mechanism of (-)-Borneol, a key herb component. Molecular docking and triplicate MD simulations suggested that (-)-Borneol engages seven distinct ORs through a conserved hydrogen-bonding anchor and adaptable hydrophobic contacts, which may explain its broad-spectrum activity in silico. The consistency between experimental potency (logEC_50_) and MM/GBSA binding free energies supports the reliability of our computational predictions. Together, these molecular findings provide a preliminary framework for understanding the potential mechanism of TCM-OT, though the causal link between broad OR activation and clinical outcomes remains hypothetical at this stage.

Previously, the benefits of OT have been attributed to its ability to promote olfactory mucosal regeneration and neuronal renewal [48, 51]. Our molecular findings offer a potential upstream explanation for these regenerative effects: the superior physicochemical diversity of TCM herb odorants enables broad and repeated activation of multiple OR families, including highly promiscuous receptors. We therefore hypothesize that this extensive receptor-level engagement may serve as an enhanced signal to trigger or amplify neuroregenerative and plastic processes, providing a possible molecular rationale for the efficacy of TCM-OT. However, this hypothetical cascade from OR activation to clinical neuroregeneration requires direct experimental validation.

Limitations of this study should be noted. Clinically, the study employed a non-randomized, non-blinded design. Control group data collection began early but proceeded more gradually; sufficient sample size for between-group comparison was achieved only recently. Differential attrition bias was observed, with higher dropout in the herb group (26/62, 41.9%) than in the control group (4/41, 9.8%), reflecting poor compliance due to low awareness of olfactory dysfunction and the greater complexity of TCM-OT. Patients who completed the study may represent a more motivated population, potentially overestimating treatment efficacy. Blinding was not feasible due to differences in intervention appearance and administration. The relatively small sample size (36 vs. 37) and lack of formal power analysis limit statistical power for secondary outcomes. Both groups received 12-week treatment, but the single-center design and predominance of post-infectious OD (61-70%) may limit generalizability. At the mechanistic level, while our in vitro luciferase assays reliably measure OR activation, they do not capture downstream signaling events. The direct causal link between broad OR engagement and in vivo neuroregeneration remains to be validated in animal models. Additionally, public databases may have incomplete coverage and platform-dependent variability, though the consistent superiority of TCM odorants in both experimental and combined datasets strengthens the conclusion.

In summary, this study provides a multi-level characterization of TCM-OT, from clinical observation to receptor-level mechanisms. The broad activation of OR families by herb odorants establish a molecular rationale for the potential efficacy of TCM-OT, while the clinical results, though preliminary, suggest a promising direction for OD treatment. Future research should therefore focus on deconvoluting this complexity to identify the minimal combination of key active compounds responsible for the optimal activation profile. Larger, multicenter randomized controlled trials with double-blind designs and extended follow-up are needed to confirm clinical efficacy and address the limitations of the present study. The identification and optimization of the key active compounds within these herbs could translate this complex bioactivity into standardized, potent formulations. This approach is essential for creating evidence-based therapeutics for olfactory rehabilitation with clear clinical and commercial translational potential.

## Supplementary Information

Additional file 1

## Data Availability

Anonymised data that support the findings of this study are available to interested academic investigators from the corresponding author (ygliu0208@163.com) upon reasonable request, after approval of a proposal and with a signed data access agreement.

## Abbreviations

CAS: Chemical Abstracts Service
CRE: CAMP response element
DVB/CAR/PDMS: Divinylbenzene/Carboxen/Polydimethylsiloxane
EC_50_: Half-maximal effective concentration
Emax: Maximum efficacy
FL: Firefly luciferase
GC-MS: Gas chromatography-mass spectrometry
GPCR: G protein-coupled receptor
HEK293T: Human embryonic kidney 293 T cells
M3R: M3 muscarinic receptor
MD: Molecular dynamics
MM/GBSA: Molecular Mechanics Generalized-Born Surface Area
NIST: National Institute of Standards and Technology
NPT: Isothermal-isobaric ensemble
NVT: Canonical ensemble
OD: Olfactory dysfunction
OIT: Olfactory Identification Test
OR: Olfactory receptor
OT: Olfactory training
PCA: Principal component analysis
QOD: Questionnaire of Olfactory Disorders
RL: Renilla luciferase
RMSD: Root-mean-square deviation
RTP1S: Receptor transporting protein 1S
SD: Standard deviation
SPME: Solid-phase microextraction
TCM: Traditional Chinese Medicine
