# Supplementary material for: A clinical and mechanistic study on traditional Chinese medicine herbs used in olfactory training
Source: Chin Med. 2026 Jun 2;21:156. doi: 10.1186/s13020-026-01432-x (PMC13227741; doi:10.1186/s13020-026-01432-x)
Supplement: Supplementary file 1 — Additional file 1 [file 13020_2026_1432_MOESM1_ESM.docx]

Supporting Figures and Tables

**
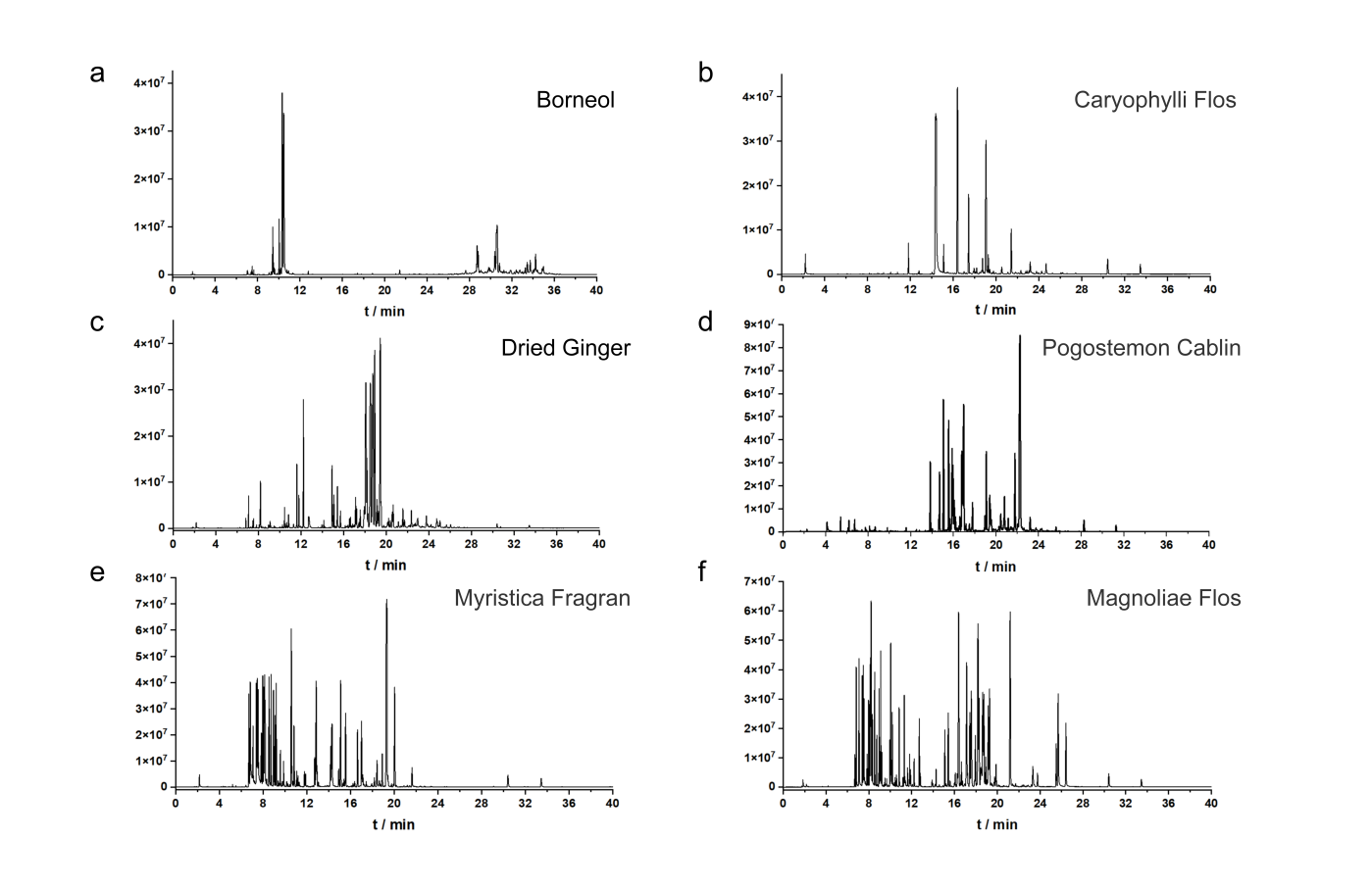
**

**Figure S1.** GC-MS total ion chromatograms of six TCM samples. (a) Borneol. (b) Caryophylli Flos. (c) Dried Ginger. (d) Pogostemon Cablin. (e) Myristica Fragran. (f) Magnoliae Flos.

**
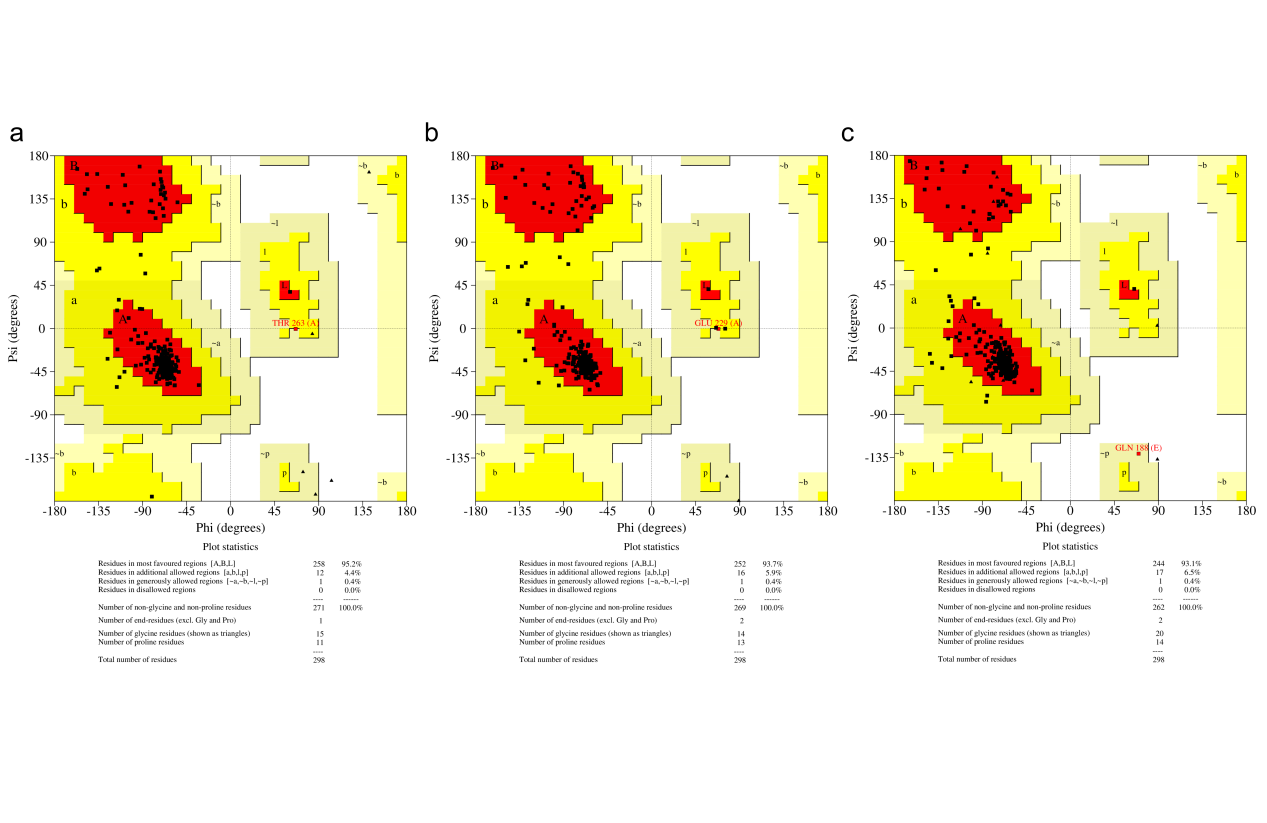
**

**Figure S2.** Ramachandran plot of the homology model showing the distribution of backbone dihedral angles (φ and ψ). (a) OR2M3. (b) OR2T11. (c) OR52L1.

**
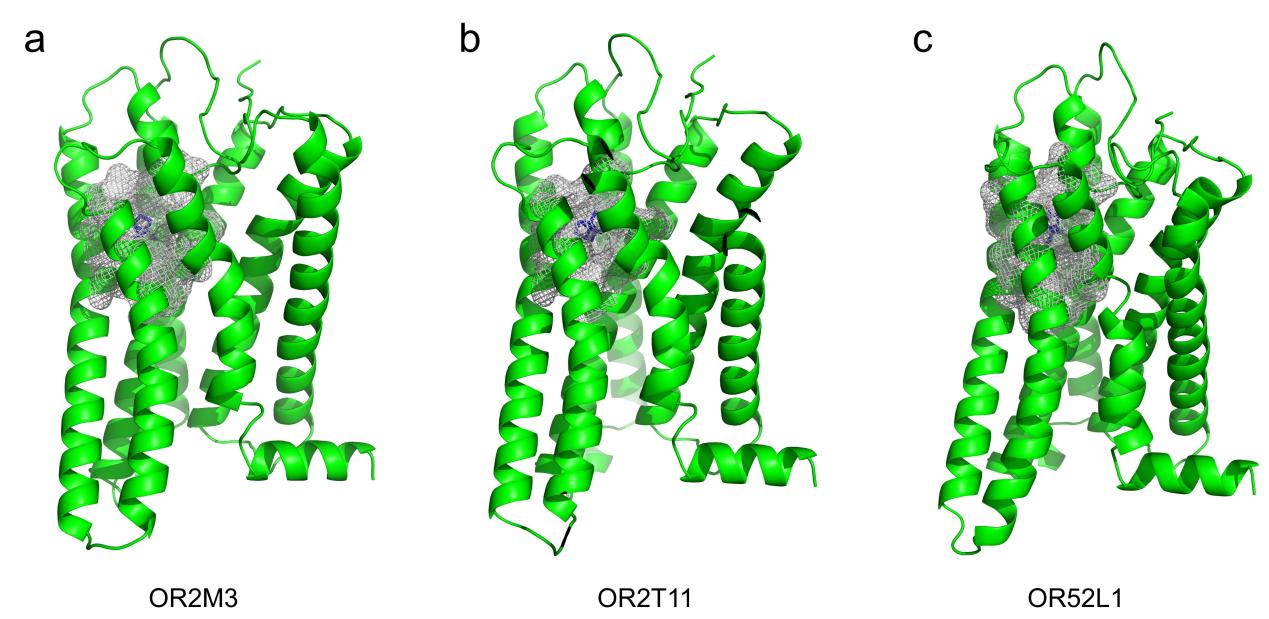
**

**Figure S3.** Binding pocket prediction using the DoGSite3 algorithm. The highest-ranked pocket (top-ranked cavity, shown in gray mesh) matches the molecular docking results (blue).

**
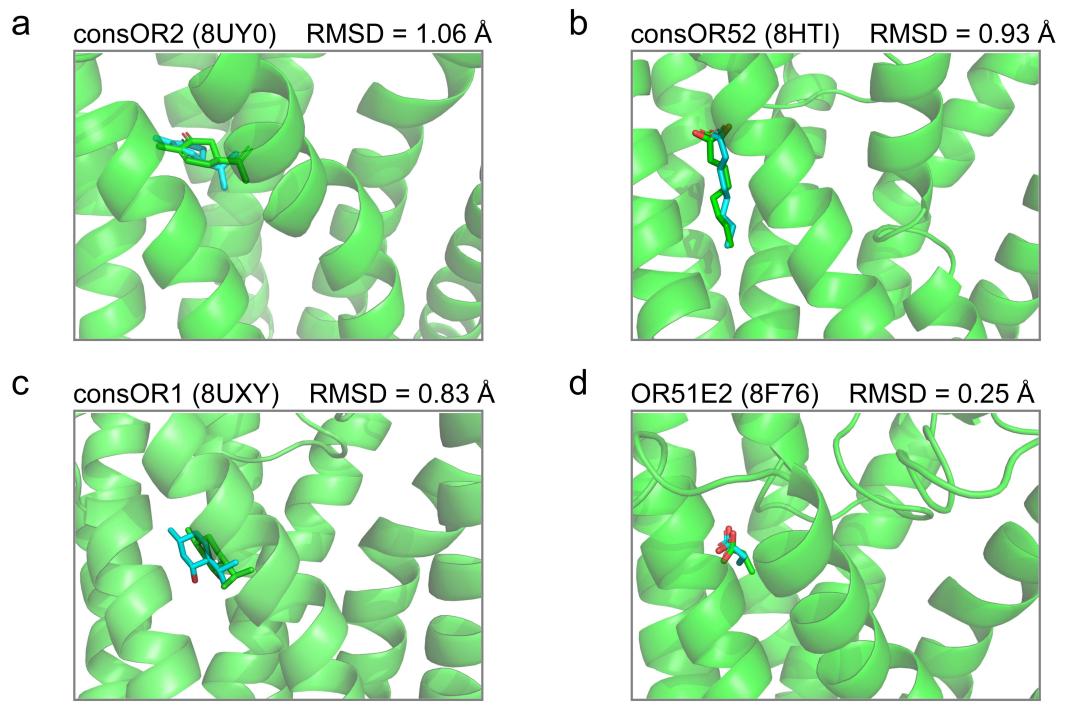
**

**Figure S4.** Re-docking validation of consOR2 (PDB id: 8UY0), consOR52 (PDB id: 8HTI), consOR1 (PDB id: 8UXY), and OR51E2 (PDB id: 8F76) showing the superimposition of crystal ligand (green) and docked ligand (cyan) with RMSD values of 1.06 Å, 0.93 Å, 0.83 Å, and 1.25 Å, respectively.


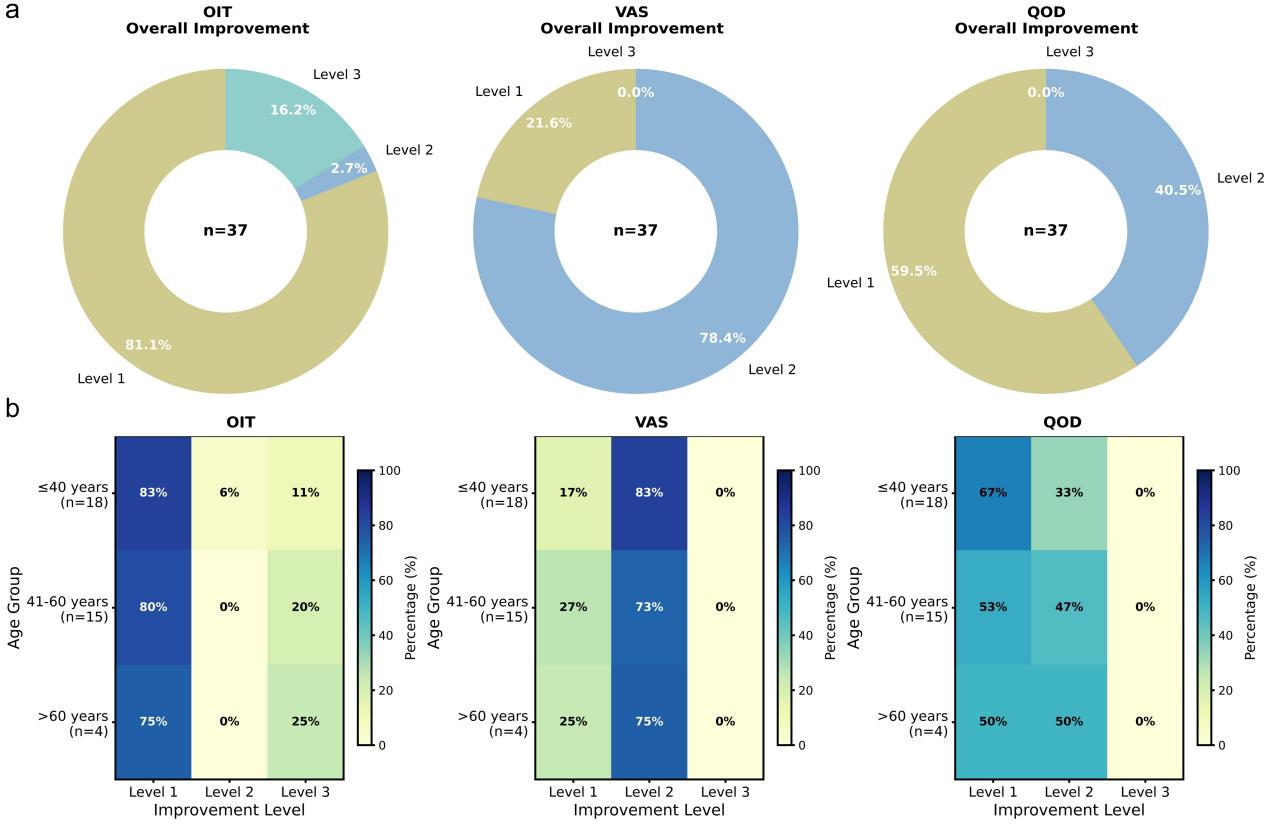


**Figure S5.** Efficacy assessment of mometasone furoate nasal spray treated (control group). (a) Overall improvement: Pie charts showing the proportion of patients achieving different levels of improvement (Level 1: slight; Level 2: moderate; Level 3: significant) for each outcome measure (OIT, VAS and QOD) across all patients. (b) Improvement by age: Heatmaps showing the distribution of improvement levels for OIT, VAS and QOD scores among patients of different ages following the therapeutic intervention.


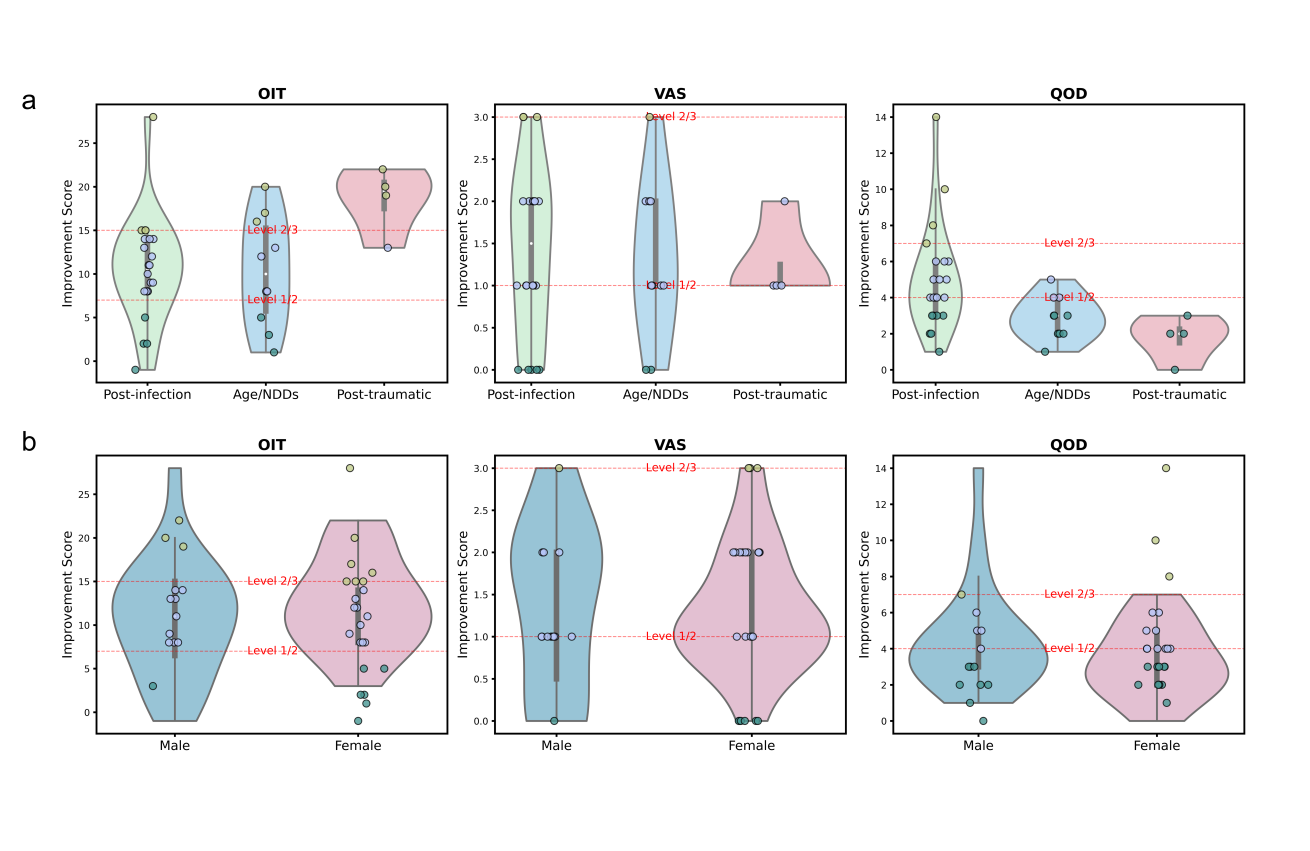


**Figure S6.** Assessment of TCM-OT outcomes by patient subgroups. (a) Improvement by olfactory dysfunction subtype: Violin plots showing the distribution of improvement scores for OIT, VAS, and QOD across different types of olfactory disorders. Red dashed lines indicate the clinical improvement levels. (b) Improvement by gender: Violin plots displaying the distribution of improvement scores for OIT, VAS, and QOD, stratified by gender. Red dashed lines indicate the clinical improvement levels.


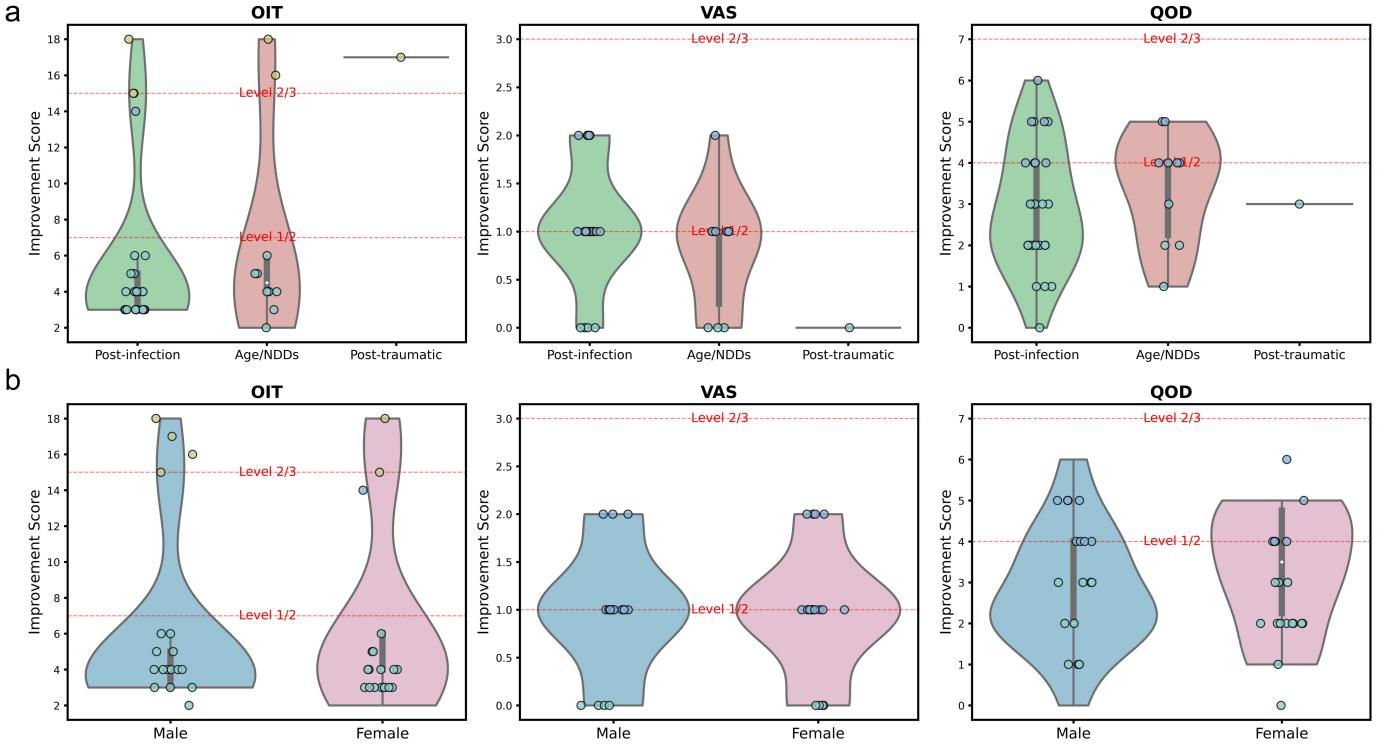


**Figure S7.** Assessment of mometasone furoate nasal spray treated (control group) outcomes by patient subgroups. (a) Improvement by olfactory dysfunction subtype: Violin plots showing the distribution of improvement scores for OIT, VAS, and QOD across different types of olfactory disorders. Red dashed lines indicate the clinical improvement levels. (b) Improvement by gender: Violin plots displaying the distribution of improvement scores for OIT, VAS, and QOD, stratified by gender. Red dashed lines indicate the clinical improvement levels.

**
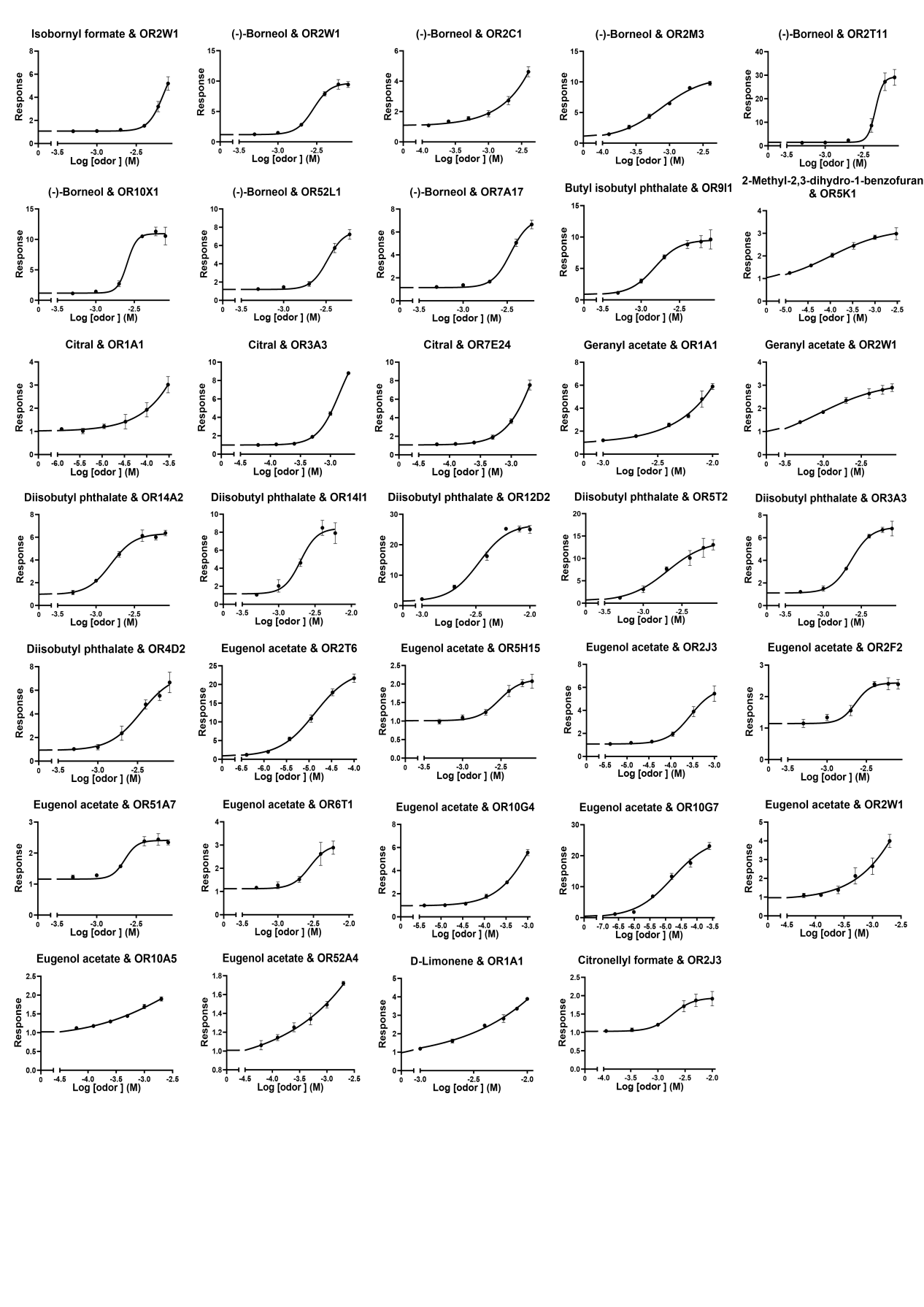
**

**Figure S8.** Responses of hORs on TCM herb odorants as measured by luciferase assay. *N = 3*.


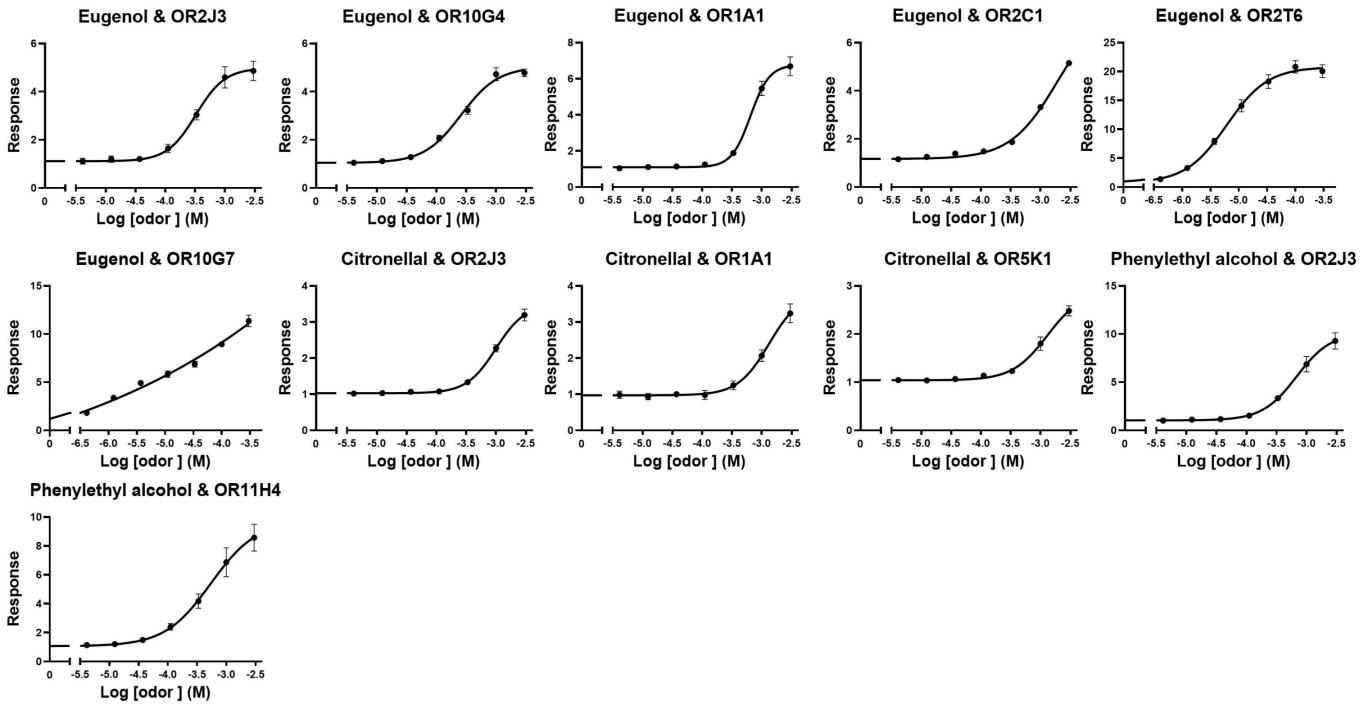


**Figure S9.** Responses of hORs on chemical odorants as measured by luciferase assay. *N = 3*.

**
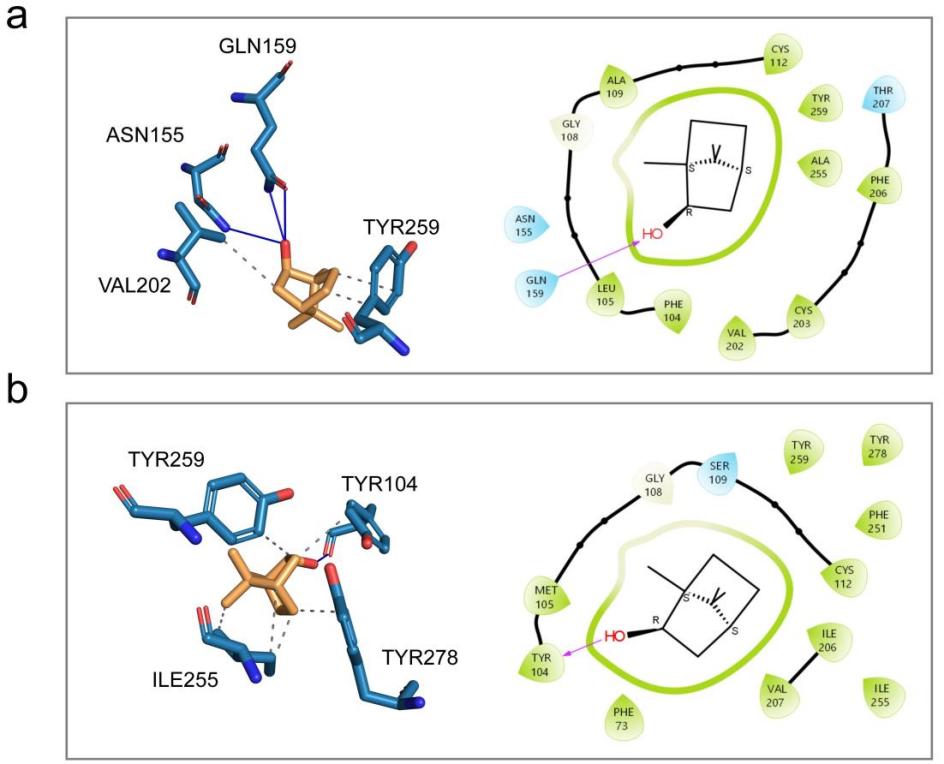

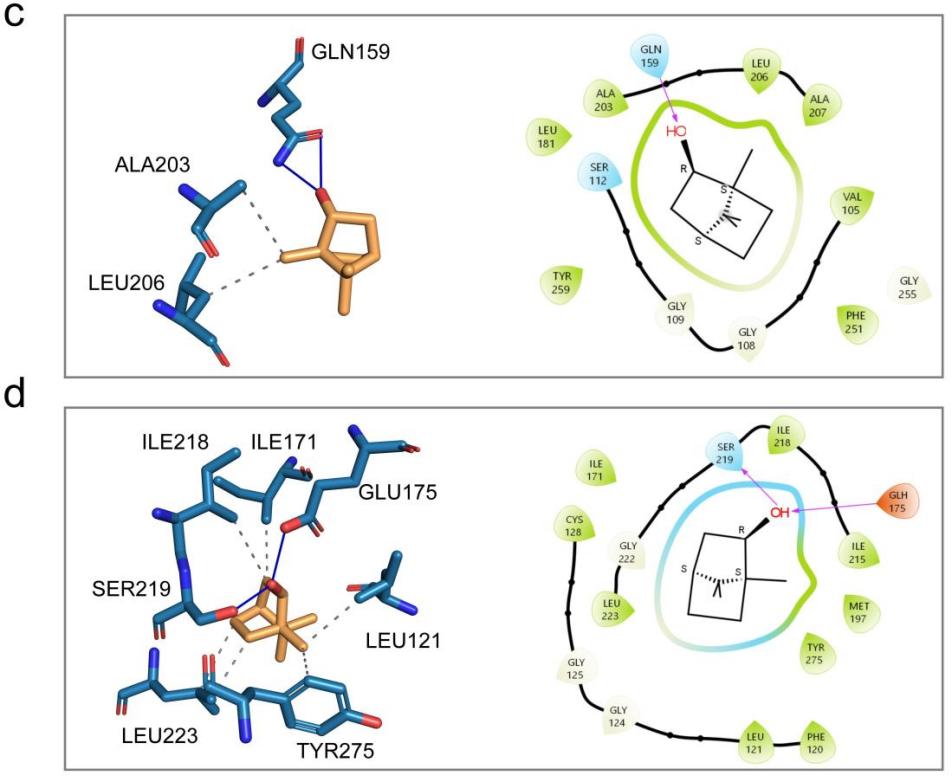
**

**Figure S10.** Molecular docking of (-)-Borneol with remaining 4 hORs. Structural models of (-)-Borneol bound to the orthosteric pockets of OR2C1 (a), OR2W1 (b), OR7A17 (c), and OR10X1 (d).


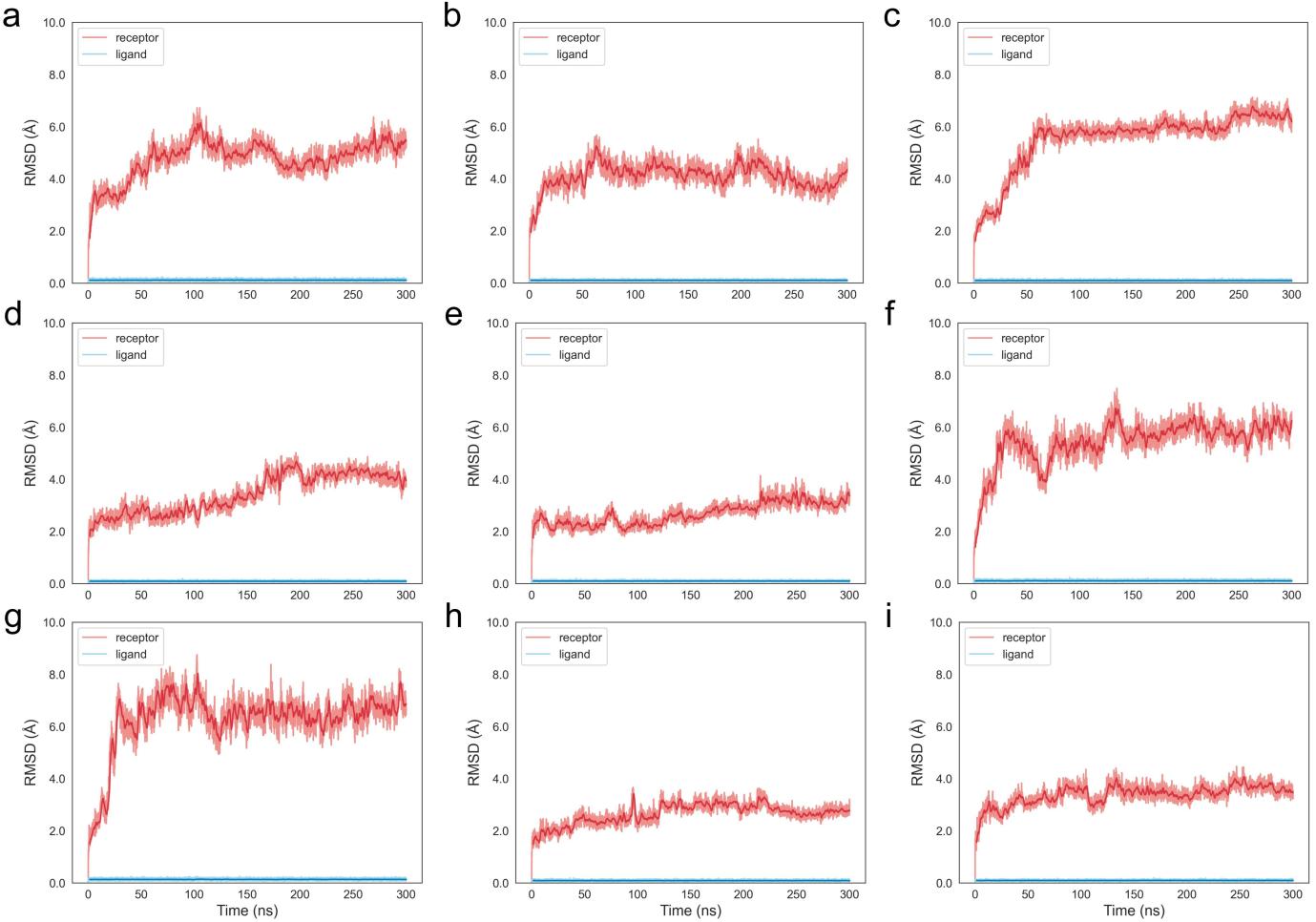


**Figure S11.** RMSD plots of MD simulations of (-)-Borneol and OR2M3 (a-c), OR2T11 (d-f), OR52L1 (g-i) complexes in independent replicate experiments.

**Table S1.** Identification of volatile components in *Borneol* by GC-MS.

| No. | t_r_/min | Chemical formula | Name | CAS | Peak area percentage (%) |
| --- | --- | --- | --- | --- | --- |
| 1 | 7.50 | C_10_H_18_ | Isohydrocamphene | 473-19-8 | 0.24 |
| 2 | 9.30 | C_10_H_18_O | Dihydrocarveol | 38049-26-2 | 0.11 |
| 3 | 9.44 | C_10_H_18_O | (-)-borneol | 464-45-9 | 2.55 |
| 4 | 10.29 | C_10_H_18_O | Borneol | 507-70-0 | 0.003 |
| 5 | 10.35 | C_11_H_18_O_2_ | Isobornyl formate | 1200-67-5 | 6.44 |

**Table S2.** Identification of volatile components in *Magnoliae Flos* by GC-MS.

| No. | t_r_/min | Chemical formula | Name | CAS | Peak area percentage (%) |
| --- | --- | --- | --- | --- | --- |
| 1 | 6.82 | C_10_H_16_ | 3-Carene | 13466-78-9 | 5.12 |
| 2 | 7.37 | C_10_H_16_ | β-Phellandrene | 555-10-2 | 2.34 |
| 3 | 7.47 | C_10_H_16_ | D-Limonene | 5989-27-5 | 4.18 |
| 4 | 7.96 | C_10_H_16_ | 3-methyl-6-(1-methylethylidene)cyclohexene | 586-63-0 | 2.39 |
| 5 | 8.32 | C_10_H_16_ | trans-β-ocimene | 3779-61-1 | 1.58 |
| 6 | 8.94 | C_10_H_16_ | Allocymene | 3016-19-1 | 2.27 |
| 7 | 10.37 | C_10_H_18_O | α-Terpineol | 10482-56-1 | 0.06 |
| 8 | 11.18 | C_12_H_20_O_2_ | 1-terpinen-4-yl acetate | 4821-04-09 | 0.14 |
| 9 | 11.30 | C_11_H_20_O_2_ | Citronellyl formate | 105-85-1 | 2.76 |
| 10 | 11.61 | C_10_H_16_O | Citral | 5392-40-5 | 0.28 |
| 11 | 12.71 | C_13_H_22_O_2_ | Isobornyl propionate | 2756-56-1 | 1.56 |
| 12 | 13.89 | C_15_H_24_ | γ-Elemene | 29873-99-2 | 0.11 |
| 13 | 14.27 | C_15_H_24_ | (±)- β-Copaene | 18252-44-3 | 0.34 |
| 14 | 14.57 | C_15_H_24_ | α-Guaiene | 3691-12-01 | 0.05 |
| 15 | 15.08 | C_15_H_24_ | α-Copaene | 3856-25-5 | 0.002 |
| 16 | 16.07 | C_15_H_24_ | (z,e)- α-Farnesene | 26560-14-5 | 0.26 |
| 17 | 16.37 | C_15_H_24_ | Caryophyllene | 13877-93-5 | 9.65 |
| 18 | 16.55 | C_15_H_24_ | (+)-bicyclogermacrene | 24703-35-3 | 0.08 |
| 19 | 17.56 | C_15_H_24_ | α-Bulnesene | 3691-11-0 | 2.69 |
| 20 | 17.94 | C_15_H_24_ | γ-Cadinene | 39029-41-9 | 1.29 |
| 21 | 18.29 | C_15_H_24_ | α-Farnesene | 502-61-4 | 1.89 |
| 22 | 23.31 | C_17_H_28_O_2_ | Guaiac acetate | 134-28-1 | 0.60 |

**Table S3.** Identification of volatile components in *Caryophylli Flo*s by GC-MS.

| No. | t_r_/min | Chemical formula | Name | CAS | Peak area percentage (%) |
| --- | --- | --- | --- | --- | --- |
| 1 | 8.31 | C_10_H_16_ | (E)-β-Ocimene | 3779-61-1 | 0.03 |
| 2 | 9.80 | C_10_H_20_O_2_ | 2-Ethylhexyl acetate | 103-09-3 | 0.005 |
| 3 | 9.89 | C_7_H_6_O_2_ | Benzoic acid | 65-85-0 | 0.015 |
| 4 | 11.81 | C_9_H_10_O | 2-Methyl-2,3-dihydro-1-benzofuran | 1746-11-8 | 3.22 |
| 5 | 12.45 | C_11_H_10_O_2_ | Vinyl trans-cinnamate | 17719-70-9 | 0.01 |
| 6 | 14.01 | C_11_H_12_O_2_ | 3-(4-Acetoxyphenyl)-1-propene | 61499-22-7 | 0.24 |
| 7 | 14.29 | C_10_H_12_O_2_ | Eugenol | 97-53-0 | 0.02 |
| 8 | 15.09 | C_15_H_24_ | (-)-(1S,4R,5R)-Guaia-6,9-dien | 36577-33-0 | 3.47 |
| 9 | 15.50 | C_8_H_8_O_3_ | Isovanillin | 621-59-0 | 0.16 |
| 10 | 16.15 | C_15_H_24_ | γ-Selinene | 515-17-3 | 0.056 |
| 11 | 16.15 | C_15_H_24_ | Valencene | 4630-07-3 | 0.08 |
| 12 | 17.01 | C_12_H_14_O_3_ | Eugenol acetate | 93-28-7 | 0.34 |
| 13 | 17.94 | C_15_H_24_ | γ-Cadinene | 39029-41-9 | 0.32 |
| 14 | 18.07 | C_15_H_22_ | (+)-Cuparene | 16982-00-6 | 0.26 |
| 15 | 18.65 | C_15_H_24_ | α-Cadinene | 24406-05-1 | 0.426 |
| 16 | 18.73 | C_15_H_24_ | 6-methyl-2-(4-methylcyclohex-3-enyl)hept-2,5-diene | 17627-44-0 | 1.87 |
| 17 | 18.86 | C_12_H_27_O_4_P | Phosphoric Acid Tris(2-methylpropyl) Ester | 126-71-6 | 0.18 |
| 18 | 21.86 | C_16_H_34_ | Hexadecane | 544-76-3 | 0.05 |
| 19 | 23.18 | C_15_H_24_O | 4(15),5,10(14)-Germacratrien-1-ol | 81968-62-9 | 1.65 |
| 20 | 30.40 | C_16_H_22_O_4_ | Butyl isobutyl phthalate | 17851-53-5 | 3.73 |
| 21 | 31.91 | C_16_H_22_O_4_ | Dibutyl phthalate | 84-74-2 | 0.02 |

**Table S4.** Identification of volatile components in *Myristica fragran* by GC-MS.

| No. | t_r_/min | Chemical formula | Name | CAS | Peak area percentage (%) |
| --- | --- | --- | --- | --- | --- |
| 1 | 6.03 | C_10_H_16_ | (1S)-(-)-alpha-Pinene | 7785-26-4 | 0.002 |
| 2 | 6.38 | C_10_H_16_ | 2-Carene | 4497-92-1 | 0.004 |
| 3 | 6.81 | C_10_H_16_ | 1-methylene-4-(1-methylvinyl) cyclohexane | 499-97-8 | 6.45 |
| 4 | 6.82 | C_10_H_16_ | 3-Carene | 13466-78-9 | 0.05 |
| 5 | 7.31 | C_10_H_16_ | α-Terpinene | 99-86-5 | 0.01 |
| 6 | 7.82 | C_10_H_16_ | γ-Terpinene | 99-85-4 | 1.67 |
| 7 | 8.12 | C_10_H_16_ | Terpinolene | 586-62-9 | 0.004 |
| 8 | 8.32 | C_10_H_16_ | (E)-β-Ocimene | 3779-61-1 | 0.07 |
| 9 | 8.73 | C_10_H_18_O | Trans-4-(isopropyl)-1-methylcyclohex-2-en-1-ol | 29803-81-4 | 2.35 |
| 10 | 9.58 | C_10_H_18_O | γ-Terpineol | 586-81-2 | 0.51 |
| 11 | 9.87 | C_10_H_18_O | trans-6-(isopropyl)-3-methylcyclohex-2-en-1-ol | 16721-39-4 | 0.35 |
| 12 | 11.19 | C_12_H_20_O_2_ | 1-Terpinen-4-yl acetate | 4821-04-9 | 0.17 |
| 13 | 11.30 | C_11_H_20_O_2_ | Citronellyl formate | 105-85-1 | 0.07 |
| 14 | 12.85 | C_10_H_10_O_2_ | Isosafrole | 120-58-1 | 4.94 |
| 15 | 14.14 | C_13_H_24_O_2_ | Citronellol propanoate | 141-14-0 | 0.25 |
| 16 | 14.38 | C_12_H_20_O_2_ | Geranyl acetate | 105-87-3 | 0.04 |
| 17 | 15.09 | C_15_H_24_ | β-copaene | 18252-44-3 | 5.24 |
| 18 | 15.49 | C_8_H_8_O_3_ | Isovanillin | 621-59-0 | 0.05 |
| 19 | 15.55 | C_11_H_14_O_2_ | (E)-methyl isoeugenol | 6380-24-1 | 2.60 |
| 20 | 16.07 | C_15_H_24_ | (Z,E)-α-Farnesene | 26560-14-5 | 0.02 |
| 21 | 16.38 | C_15_H_24_ | Caryophyllene | 13877-93-5 | 0.11 |
| 22 | 16.64 | C1_5_H_24_ | Zingiberene | 495-60-3 | 1.84 |
| 23 | 17.01 | C_12_H_14_O_3_ | Eugenol acetate | 93-28-7 | 2.63 |
| 24 | 17.12 | C_15_H_24_ | β-sesquiphellandrene | 20307-83-9 | 0.22 |
| 25 | 18.18 | C_15_H_24_ | γ-Cadinene | 39029-41-9 | 0.22 |
| 26 | 18.90 | C_15_H_24_ | β-bisabolene | 495-61-4 | 0.77 |
| 27 | 20.03 | C_12_H_16_O_3_ | β-Asarone | 5273-86-9 | 4.01 |
| 28 | 21.20 | C_15_H_24_O | Isospathulenol | 88395-46-4 | 0.04 |
| 29 | 30.41 | C_16_H_22_O_4_ | Butyl isobutyl phthalate | 17851-53-5 | 0.77 |

**Table S5.** Identification of volatile components in *Dried ginger* by GC-MS.

| No. | t_r_/min | Chemical formula | Name | CAS | Peak area percentage (%) |
| --- | --- | --- | --- | --- | --- |
| 1 | 6.66 | C_10_H_16_ | (1S)-(+)-3-Carene | 498-15-7 | 0.01 |
| 2 | 6.82 | C_10_H_16_ | Ocimene mixture of isomers | 3338-55-4 | 0.2 |
| 3 | 7.08 | C_10_H_16_ | 3-Carene | 13466-78-9 | 0.66 |
| 4 | 7.37 | C_10_H_16_ | α-Phellandrene | 99-83-2 | 0.01 |
| 5 | 7.55 | C_10_H_16_ | β-Phellandrene | 555-10-2 | 0.19 |
| 6 | 7.96 | C_10_H_16_ | Terpinolene | 586-62-9 | 0.01 |
| 7 | 8.14 | C_10_H_16_ | (+)-Dipentene | 5989-27-5 | 0.26 |
| 8 | 8.17 | C_10_H_16_ | γ-Terpinene | 99-85-4 | 1.45 |
| 9 | 8.32 | C_10_H_16_ | (E)-β-Ocimene | 3779-61-1 | 0.05 |
| 10 | 9.10 | C_11_H_18_O_2_ | Linalyl formate | 115-99-1 | 0.15 |
| 11 | 10.85 | C_10_H_12_O | (Z)-Anethole | 25679-28-1 | 0.28 |
| 12 | 11.29 | C_11_H_20_O_2_ | Citronellyl formate | 105-85-1 | 0.12 |
| 13 | 12.22 | C_10_H_16_O | Citral | 5392-40-5 | 5.3 |
| 14 | 14.14 | C_12_H_22_O_2_ | Citronellyl acetate | 150-84-5 | 0.28 |
| 15 | 14.27 | C_15_H_24_ | β-Cubebene | 13744-15-5 | 0.04 |
| 16 | 14.91 | C_12_H_20_O_2_ | Geranyl acetate | 105-87-3 | 2.53 |
| 17 | 15.09 | C_15_H_24_ | (±)-β-Copaene | 18252-44-3 | 1.41 |
| 18 | 15.49 | C_8_H_8_O_3_ | Isovanillin | 621-59-0 | 0.09 |
| 19 | 15.70 | C_15_H_24_ | (E)-α-bergamotene,(-)-trans-α-bergamotene | 13474-59-4 | 0.51 |
| 20 | 16.54 | C_15_H_24_ | (+)-Bicyclogermacrene | 24703-35-3 | 0.37 |
| 21 | 16.85 | C_9_H_10_O_3_ | Acetovanillone | 498-02-2 | 0.19 |
| 22 | 17.12 | C_15_H_24_ | β-Sesquiphellandrene | 20307-83-9 | 1.26 |
| 23 | 17.42 | C_15_H_24_ | α-Bulnesene | 3691-11-0 | 0.16 |
| 24 | 18.11 | C_15_H_22_ | α-Curcumene | 644-30-4 | 0.01 |
| 25 | 18.29 | C_15_H_24_ | α-Cadinene | 24406-05-1 | 0.65 |
| 26 | 18.75 | C_15_H_24_ | γ- Cadinene | 39029-41-9 | 13.1 |
| 27 | 20.22 | C_15_H_26_O | Elemol | 639-99-6 | 0.42 |
| 28 | 20.65 | C_15_H_24_ | GermacreneB | 15423-57-1 | 1.09 |

**Table S6.** Identification of volatile components in *Pogostemon cablin* by GC-MS.

| No. | t_r_/min | Chemical formula | Name | CAS | Peak area percentage (%) |
| --- | --- | --- | --- | --- | --- |
| 1 | 5.42 | C_8_H_8_ | Styrene | 100-42-5 | 0.005 |
| 2 | 5.95 | C_10_H_16_ | α-Thujene | 2867-05-2 | 0.001 |
| 3 | 6.08 | C_10_H_16_ | α-Pinene | 80-56-8 | 0.09 |
| 4 | 6.49 | C_7_H_6_O | Benzaldehyde | 100-52-7 | 0.04 |
| 5 | 6.64 | C_10_H_16_ | Sabinen | 3387-41-5 | 0.24 |
| 7 | 6.84 | C_10_H_16_ | Myrcene | 123-35-3 | 0.007 |
| 8 | 7.24 | C_10_H_16_ | α-Terpinene | 99-86-5 | 0.001 |
| 9 | 7.40 | C_10_H_16_ | D-Limonene | 5989-27-5 | 0.05 |
| 10 | 7.76 | C_10_H_16_ | γ-Terpinene | 99-85-4 | 0.002 |
| 11 | 8.27 | C_10_H_18_O | Linalool | 78-70-6 | 0.02 |
| 12 | 9.75 | C_10_H_18_O | α-Terpineol | 10482-56-1 | 0.01 |
| 13 | 9.78 | C_10_H_12_O | Estragole | 140-67-0 | 0.13 |
| 14 | 9.80 | C_10_H_16_O | Dihydrocarvone | 5524-05-0 | 0.01 |
| 15 | 10.19 | C_10_H_20_O | Citronellol | 106-22-9 | 0.01 |
| 16 | 10.28 | C_11_H_16_O | [2-isopropyl-4-methyl anisole](https://www.chemsrc.com/en/cas/31574-44-4_1153310.html) | 31574-44-4 | 0.01 |
| 17 | 10.97 | C_9_H_12_O_2_ | 3,5-Dimethoxytoluene | 4179-19-5 | 0.01 |
| 18 | 11.43 | C_10_H_12_O | Anethole | 104-46-1 | 0.02 |
| 19 | 12.52 | C_15_H_24_ | δ-Elemene | 20307-84-0 | 0.10 |
| 20 | 13.81 | C_15_H_24_ | β-Maaliene | 489-29-2 | 5.56 |
| 21 | 15.05 | C_15_H_24_ | α-Guaiene | 3691-12-01 | 20.9 |
| 22 | 15.66 | C_15_H_24_ | 1-methyl-4-(1-methylethylidene)-2-(1-methylvinyl)-1-vinylcyclohexane | 3242-08-08 | 0.78 |
| 23 | 15.87 | C_15_H_24_ | 1H-Indene,2,4,5,6,7,7a-hexahydro-3,7-dimethyl-4-(2-methyl-1-propen-1-yl)-, (4S,7R,7aR)- | 351222-66-7 | 8.00 |
| 24 | 15.95 | C_15_H_24_ | (+)-γ-Gurjunene | 22567-17-5 | 5.36 |
| 25 | 16.07 | C_15_H_24_ | Patchoulene | 1405-16-9 | 1.85 |
| 26 | 16.60 | C_15_H_24_ | cis-β-Guaiene | 87745-31-1 | 0.99 |
| 27 | 16.77 | C_15_H_24_ | Valencene | 4630-07-03 | 7.17 |
| 28 | 18.94 | C_15_H_24_O | Spathulenol | 6750-60-3 | 0.98 |
| 29 | 21.13 | C15H26O | Pogostol | 21698-41-9 | 0.87 |
| 30 | 23.53 | C12H16O4 | Pogostone | 23800-56-8 | 0.02 |
| 31 | 28.24 | C16H22O4 | Diisobutyl phthalate | 84-69-5 | 1.43 |
| 32 | 31.25 | C16H22O4 | Butyl isobutyl phthalate | 17851-53-5 | 0.81 |

**Table S7.** Identification of major components in six TCM herbs using GC-MS (peak area > 0.05%, match factor ≥ 85)

| No. | Name | CAS | Chemical formula |
| --- | --- | --- | --- |
| 1 | α-Guaiene | 3691-12-01 | C15H24 |
| 2 | 1H-Indene,2,4,5,6,7,7a-hexahydro-3,7-dimethyl-4-(2-methyl-1-propen-1-yl)-,(4S,7R,7aR)- | 351222-66-7 | C15H24 |
| 3 | Valencene | 4630-07-3 | C15H24 |
| 4 | 1-methylene-4-(1-methylvinyl)cyclohexane | 499-97-8 | C10H16 |
| 5 | isobornyl formate | 1200-67-5 | C11H18O2 |
| 7 | β-Maaliene | 489-29-2 | C15H24 |
| 8 | (+)-γ-Gurjunene | 22567-17-5 | C15H24 |
| 9 | Citral | 5392-40-5 | C10H16O |
| 10 | Isosafrole | 120-58-1 | C10H10O2 |
| 11 | β-Asarone | 5273-86-9 | C12H16O3 |
| 12 | (-)-(1S,4R,5R)-Guaia-6,9-dien | 36577-33-0 | C15H24 |
| 13 | 2-Methyl-2,3-dihydro-1-benzofuran | 1746-11-8 | C9H10O |
| 14 | (E)-methylisoeugenol | 6380-24-1 | C11H14O2 |
| 15 | (-)-Borneol | 464-45-9 | C10H18O |
| 16 | Geranylacetate | 105-87-3 | C12H20O2 |
| 17 | 3-methyl-6-(1-methylethylidene)cyclohexene | 586-63-0 | C10H16 |
| 18 | Trans-4-(isopropyl)-1-methylcyclohex-2-en-1-ol | 29803-81-4 | C10H18O |
| 19 | Allocymene | 3016-19-1 | C10H16 |
| 20 | α-Farnesene | 502-61-4 | C15H24 |
| 21 | 6-methyl-2-(4-methylcyclohex-3-enyl)hept-2,5-diene | 17627-44-0 | C15H24 |
| 22 | Patchoulene | 1405-16-9 | C15H24 |
| 23 | Zingiberene | 495-60-3 | C15H24 |
| 24 | 4(15),5,10(14)-Germacratrien-1-ol | 81968-62-9 | C15H24O |
| 25 | Isobornylpropionate | 2756-56-1 | C13H22O2 |
| 26 | γ-Terpinene | 99-85-4 | C10H16 |
| 27 | Diisobutylphthalate | 84-69-5 | C16H22O4 |
| 28 | (±)-β-Copaene | 18252-44-3 | C15H24 |
| 29 | β-Sesquiphellandrene | 20307-83-9 | C15H24 |
| 30 | GermacreneB | 15423-57-1 | C15H24 |
| 31 | cis-β-Guaiene | 87745-31-1 | C15H24 |
| 32 | Spathulenol | 6750-60-3 | C15H24O |
| 33 | Pogostol | 21698-41-9 | C15H26O |
| 34 | Butylisobutylphthalate | 17851-53-5 | C16H22O4 |
| 35 | 1-methyl-4-(1-methylethylidene)-2-(1-methylvinyl)-1-vinylcyclohexane | 3242-08-08 | C15H24 |
| 36 | β-bisabolene | 495-61-4 | C15H24 |
| 37 | 3-Carene | 13466-78-9 | C10H16 |
| 38 | Guaiacacetate | 134-28-1 | C17H28O2 |
| 39 | (E)-α-bergamotene,(-)-trans-α-bergamotene | 13474-59-4 | C15H24 |
| 40 | γ-Terpineol | 586-81-2 | C10H18O |
| 41 | α-Cadinene | 24406-05-1 | C15H24 |
| 42 | Elemol | 639-99-6 | C15H26O |
| 43 | (+)-Bicyclogermacrene | 24703-35-3 | C15H24 |
| 44 | trans-6-(isopropyl)-3-methylcyclohex-2-en-1-ol | 16721-39-4 | C10H18O |
| 45 | Eugenolacetate | 93-28-7 | C12H14O3 |
| 46 | γ-Cadinene | 39029-41-9 | C15H24 |
| 47 | (Z)-Anethole | 25679-28-1 | C10H12O |
| 48 | Citronellylacetate | 150-84-5 | C12H22O2 |
| 49 | (+)-Cuparene | 16982-00-6 | C15H22 |
| 50 | (+)-Dipentene | 5989-27-5 | C10H16 |
| 51 | (z,e)-α-Farnesene | 26560-14-5 | C15H24 |
| 52 | Citronellolpropanoate | 141-14-0 | C13H24O2 |
| 53 | Sabinen | 3387-41-5 | C10H16 |
| 54 | 3-(4-Acetoxyphenyl)-1-propene | 61499-22-7 | C11H12O2 |
| 55 | Isohydrocamphene | 473-19-8 | C10H18 |
| 56 | Ocimenemixtureofisomers | 3338-55-4 | C10H16 |
| 57 | β-Phellandrene | 555-10-2 | C10H16 |
| 58 | Acetovanillone | 498-02-2 | C9H10O3 |
| 59 | PhosphoricAcidTris(2-methylpropyl)Ester | 126-71-6 | C12H27O4P |
| 60 | 1-Terpinen-4-ylacetate | 4821-04-09 | C12H20O2 |
| 61 | Isovanillin | 621-59-0 | C8H8O3 |
| 62 | α-Bulnesene | 3691-11-0 | C15H24 |
| 63 | Linalylformate | 115-99-1 | C11H18O2 |
| 64 | Estragole | 140-67-0 | C10H12O |
| 65 | Citronellylformate | 105-85-1 | C11H20O2 |
| 66 | Caryophyllene | 13877-93-5 | C15H24 |
| 67 | γ-Elemene | 29873-99-2 | C15H24 |
| 68 | Dihydrocarveol | 38049-26-2 | C10H18O |
| 69 | δ-Elemene | 20307-84-0 | C15H24 |
| 70 | α-Pinene | 80-56-8 | C10H16 |
| 71 | (E)-β-Ocimene | 3779-61-1 | C10H16 |
| 72 | α-Terpineol | 10482-56-1 | C10H18O |

**Table S8.** All the TCM odorant-hOR pairs from this paper and databases.

| **No.** | **Odorant** | **OR** | **Source** |
| --- | --- | --- | --- |
| 1 | Eugenol acetate | OR10G7 | this paper; M2OR; OlfactionBase |
| 2 | Geranyl acetate | OR2W1 | this paper; M2OR |
| 3 | Eugenol acetate | OR2J3 | this paper; M2OR |
| 4 | Eugenol acetate | OR6T1 | this paper; M2OR |
| 5 | Eugenol acetate | OR2W1 | this paper; M2OR |
| 6 | Eugenol acetate | OR10A5 | this paper; M2OR |
| 7 | D-Limonene | OR1A1 | this paper; M2OR |
| 8 | Citral | OR1A1 | this paper; M2OR |
| 9 | Isobornyl formate | OR2W1 | this paper |
| 10 | Geranyl acetate | OR1A1 | this paper |
| 11 | Eugenol acetate | OR2T6 | this paper |
| 12 | Eugenol acetate | OR5H15 | this paper |
| 13 | Eugenol acetate | OR2F2 | this paper |
| 14 | Eugenol acetate | OR51A7 | this paper |
| 15 | Eugenol acetate | OR10G4 | this paper |
| 16 | Eugenol acetate | OR52A4 | this paper |
| 17 | Diisobutyl phthalate | OR14A2 | this paper |
| 18 | Diisobutyl phthalate | OR14I1 | this paper |
| 19 | Diisobutyl phthalate | OR12D2 | this paper |
| 20 | Diisobutyl phthalate | OR5T2 | this paper |
| 21 | Diisobutyl phthalate | OR3A3 | this paper |
| 22 | Diisobutyl phthalate | OR4D2 | this paper |
| 23 | Citronellyl formate | OR2J3 | this paper |
| 24 | Citral | OR3A3 | this paper |
| 25 | Citral | OR7E24 | this paper |
| 26 | Butyl isobutyl phthalate | OR9I1 | this paper |
| 27 | 2-Methyl-2,3-dihydro-1-benzofuran | OR5K1 | this paper |
| 28 | (-)-Borneol | OR2W1 | this paper |
| 29 | (-)-Borneol | OR2C1 | this paper |
| 30 | (-)-Borneol | OR2M3 | this paper |
| 31 | (-)-Borneol | OR2T11 | this paper |
| 32 | (-)-Borneol | OR10X1 | this paper |
| 33 | (-)-Borneol | OR52L1 | this paper |
| 34 | (-)-Borneol | OR7A17 | this paper |
| 35 | Eugenol acetate | OR10AG1 | OlfactionBase |
| 36 | Eugenol acetate | OR8D1 | OlfactionBase |
| 37 | Eugenol acetate | OR7C1 | OlfactionBase |
| 38 | Eugenol acetate | OR5B17 | OlfactionBase |
| 39 | Eugenol acetate | OR5AC2 | OlfactionBase |
| 40 | Eugenol acetate | OR2B3 | OlfactionBase |
| 41 | Eugenol acetate | OR1G1 | OlfactionBase |
| 42 | Estragole | OR2M4 | OlfactionBase |
| 43 | Eugenol acetate | OR1D5 | M2OR; OlfactionBase |
| 44 | Estragole | OR52D1 | M2OR; OlfactionBase |
| 45 | Geranyl acetate | OR2H2 | M2OR |
| 46 | Geranyl acetate | OR9A4 | M2OR |
| 47 | Geranyl acetate | OR2A14 | M2OR |
| 48 | Geranyl acetate | OR10G4 | M2OR |
| 49 | Geranyl acetate | OR51M1 | M2OR |
| 50 | Geranyl acetate | OR4D5 | M2OR |
| 51 | Geranyl acetate | OR52E4 | M2OR |
| 52 | Geranyl acetate | OR52E5 | M2OR |
| 53 | Geranyl acetate | OR5M10 | M2OR |
| 54 | Geranyl acetate | OR51E1 | M2OR |
| 55 | Geranyl acetate | OR5F1 | M2OR |
| 56 | Geranyl acetate | OR4C12 | M2OR |
| 57 | Geranyl acetate | OR2M5 | M2OR |
| 58 | Geranyl acetate | OR56B1 | M2OR |
| 59 | Geranyl acetate | OR5M9 | M2OR |
| 60 | Geranyl acetate | OR10Q1 | M2OR |
| 61 | Geranyl acetate | OR10A6 | M2OR |
| 62 | Geranyl acetate | OR52W1 | M2OR |
| 63 | Geranyl acetate | OR5M11 | M2OR |
| 64 | Geranyl acetate | OR4C16 | M2OR |
| 65 | Geranyl acetate | OR52N5 | M2OR |
| 66 | Geranyl acetate | OR6X1 | M2OR |
| 67 | Geranyl acetate | OR8B2 | M2OR |
| 68 | Geranyl acetate | OR5J2 | M2OR |
| 69 | Geranyl acetate | OR11H4 | M2OR |
| 70 | Geranyl acetate | OR4L1 | M2OR |
| 71 | Geranyl acetate | OR1D2 | M2OR |
| 72 | Geranyl acetate | OR2A25 | M2OR |
| 73 | Geranyl acetate | OR2J2 | M2OR |
| 74 | Geranyl acetate | OR6C68 | M2OR |
| 75 | Geranyl acetate | OR2C3 | M2OR |
| 76 | Geranyl acetate | OR6K3 | M2OR |
| 77 | Geranyl acetate | OR2M2 | M2OR |
| 78 | Geranyl acetate | OR51B4 | M2OR |
| 79 | Geranyl acetate | OR4F4 | M2OR |
| 80 | Geranyl acetate | OR4F17 | M2OR |
| 81 | Geranyl acetate | OR4M1 | M2OR |
| 82 | Geranyl acetate | OR2T35 | M2OR |
| 83 | Geranyl acetate | OR2A1 | M2OR |
| 84 | Geranyl acetate | OR4K15 | M2OR |
| 85 | Geranyl acetate | OR52A5 | M2OR |
| 86 | Geranyl acetate | OR52N2 | M2OR |
| 87 | Geranyl acetate | OR2T8 | M2OR |
| 88 | Geranyl acetate | OR4F21 | M2OR |
| 89 | Geranyl acetate | OR10G7 | M2OR |
| 90 | Geranyl acetate | OR2J3 | M2OR |
| 91 | Geranyl acetate | OR3A1 | M2OR |
| 92 | Geranyl acetate | OR4A5 | M2OR |
| 93 | Geranyl acetate | OR1D5 | M2OR |
| 94 | Geranyl acetate | OR1F12P | M2OR |
| 95 | Geranyl acetate | OR2L5 | M2OR |
| 96 | Geranyl acetate | OR51S1 | M2OR |
| 97 | Geranyl acetate | OR51V1 | M2OR |
| 98 | Geranyl acetate | OR52H1 | M2OR |
| 99 | Geranyl acetate | OR2T33 | M2OR |
| 100 | Geranyl acetate | OR10J1 | M2OR |
| 101 | Geranyl acetate | OR11G2 | M2OR |
| 102 | Geranyl acetate | OR6J1 | M2OR |
| 103 | Geranyl acetate | OR13C2 | M2OR |
| 104 | Geranyl acetate | OR52A4P | M2OR |
| 105 | Geranyl acetate | OR56A4 | M2OR |
| 106 | Geranyl acetate | OR8B3 | M2OR |
| 107 | Geranyl acetate | OR5K4 | M2OR |
| 108 | Geranyl acetate | OR10H2 | M2OR |
| 109 | Eugenol acetate | OR2F1 | M2OR |
| 110 | Eugenol acetate | OR13J1 | M2OR |
| 111 | Eugenol acetate | OR51M1 | M2OR |
| 112 | Eugenol acetate | OR4D5 | M2OR |
| 113 | Eugenol acetate | OR5A1 | M2OR |
| 114 | Eugenol acetate | OR5A2 | M2OR |
| 115 | Eugenol acetate | OR5I1 | M2OR |
| 116 | Eugenol acetate | OR5F1 | M2OR |
| 117 | Eugenol acetate | OR4C12 | M2OR |
| 118 | Eugenol acetate | OR52E2 | M2OR |
| 119 | Eugenol acetate | OR5P2 | M2OR |
| 120 | Eugenol acetate | OR1M1 | M2OR |
| 121 | Eugenol acetate | OR10Q1 | M2OR |
| 122 | Eugenol acetate | OR51E2 | M2OR |
| 123 | Eugenol acetate | OR2D2 | M2OR |
| 124 | Eugenol acetate | OR5M11 | M2OR |
| 125 | Eugenol acetate | OR52N5 | M2OR |
| 126 | Eugenol acetate | OR6X1 | M2OR |
| 127 | Eugenol acetate | OR8B2 | M2OR |
| 128 | Eugenol acetate | OR5J2 | M2OR |
| 129 | Eugenol acetate | OR4D2 | M2OR |
| 130 | Eugenol acetate | OR7G3 | M2OR |
| 131 | Eugenol acetate | OR1L6 | M2OR |
| 132 | Eugenol acetate | OR13C3 | M2OR |
| 133 | Eugenol acetate | OR2J2 | M2OR |
| 134 | Eugenol acetate | OR6C68 | M2OR |
| 135 | Eugenol acetate | OR2C3 | M2OR |
| 136 | Eugenol acetate | OR1S1 | M2OR |
| 137 | Eugenol acetate | OR10J3 | M2OR |
| 138 | Eugenol acetate | OR5H14 | M2OR |
| 139 | Eugenol acetate | OR3A3 | M2OR |
| 140 | Eugenol acetate | OR3A4P | M2OR |
| 141 | Eugenol acetate | OR51B2 | M2OR |
| 142 | Eugenol acetate | OR51B4 | M2OR |
| 143 | Eugenol acetate | OR51B5 | M2OR |
| 144 | Eugenol acetate | OR51B6 | M2OR |
| 145 | Eugenol acetate | OR51I1 | M2OR |
| 146 | Eugenol acetate | OR4F4 | M2OR |
| 147 | Eugenol acetate | OR4F17 | M2OR |
| 148 | Eugenol acetate | OR11H1 | M2OR |
| 149 | Eugenol acetate | OR4M1 | M2OR |
| 150 | Eugenol acetate | OR2T2 | M2OR |
| 151 | Eugenol acetate | OR2T35 | M2OR |
| 152 | Eugenol acetate | OR2A1 | M2OR |
| 153 | Eugenol acetate | OR4K15 | M2OR |
| 154 | Eugenol acetate | OR52A5 | M2OR |
| 155 | Eugenol acetate | OR52N2 | M2OR |
| 156 | Eugenol acetate | OR2T8 | M2OR |
| 157 | Eugenol acetate | OR51A2 | M2OR |
| 158 | Eugenol acetate | OR4A47 | M2OR |
| 159 | Eugenol acetate | OR4F21 | M2OR |
| 160 | Eugenol acetate | OR4A5 | M2OR |
| 161 | Eugenol acetate | OR1F12P | M2OR |
| 162 | Eugenol acetate | OR1J1 | M2OR |
| 163 | Eugenol acetate | OR2L5 | M2OR |
| 164 | Eugenol acetate | OR51J1 | M2OR |
| 165 | Eugenol acetate | OR51S1 | M2OR |
| 166 | Eugenol acetate | OR51V1 | M2OR |
| 167 | Eugenol acetate | OR52H1 | M2OR |
| 168 | Eugenol acetate | OR52R1 | M2OR |
| 169 | Eugenol acetate | OR2T33 | M2OR |
| 170 | Eugenol acetate | OR56A5 | M2OR |
| 171 | Eugenol acetate | OR10J1 | M2OR |
| 172 | Eugenol acetate | OR11G2 | M2OR |
| 173 | Eugenol acetate | OR9Q1 | M2OR |
| 174 | Eugenol acetate | OR6J1 | M2OR |
| 175 | Eugenol acetate | OR13C2 | M2OR |
| 176 | Eugenol acetate | OR2V1 | M2OR |
| 177 | Eugenol acetate | OR52A4P | M2OR |
| 178 | Eugenol acetate | OR5L1 | M2OR |
| 179 | Eugenol acetate | OR56A4 | M2OR |
| 180 | Eugenol acetate | OR5B12 | M2OR |
| 181 | Eugenol acetate | OR8B3 | M2OR |
| 182 | Eugenol acetate | OR5K4 | M2OR |
| 183 | Eugenol acetate | OR6V1 | M2OR |
| 184 | Eugenol acetate | OR8B12 | M2OR |
| 185 | Eugenol acetate | OR10G3 | M2OR |
| 186 | Eugenol acetate | OR9G9 | M2OR |
| 187 | Estragole | OR2W1 | M2OR |
| 188 | Estragole | OR1A1 | M2OR |
| 189 | Estragole | OR8H1 | M2OR |
| 190 | D-Limonene | OR2W1 | M2OR |
| 191 | Citral | OR10G4 | M2OR |
| 192 | Citral | OR51M1 | M2OR |
| 193 | Citral | OR52N4 | M2OR |
| 194 | Citral | OR51E1 | M2OR |
| 195 | Citral | OR52W1 | M2OR |
| 196 | Citral | OR2D2 | M2OR |
| 197 | Citral | OR52N5 | M2OR |
| 198 | Citral | OR1D2 | M2OR |
| 199 | Citral | OR6C68 | M2OR |
| 200 | Citral | OR2W1 | M2OR |
| 201 | Citral | OR51B2 | M2OR |
| 202 | Citral | OR51B6 | M2OR |
| 203 | Citral | OR51I1 | M2OR |
| 204 | Citral | OR52A5 | M2OR |
| 205 | Citral | OR51A2 | M2OR |
| 206 | Citral | OR2J3 | M2OR |
| 207 | Citral | OR4A5 | M2OR |
| 208 | Citral | OR1D5 | M2OR |
| 209 | Citral | OR1J1 | M2OR |
| 210 | Citral | OR52H1 | M2OR |
| 211 | Citral | OR2T33 | M2OR |
| 212 | Citral | OR10A5 | M2OR |
| 213 | alpha-Terpineol | OR2W1 | M2OR |
| 214 | alpha-Terpineol | OR52N5 | M2OR |
| 215 | alpha-Terpineol | OR6X1 | M2OR |
| 216 | alpha-Terpineol | OR8B2 | M2OR |
| 217 | alpha-Terpineol | OR1A1 | M2OR |
| 218 | alpha-Terpineol | OR51B6 | M2OR |
| 219 | alpha-Terpineol | OR51I1 | M2OR |
| 220 | alpha-Terpineol | OR4F4 | M2OR |
| 221 | alpha-Terpineol | OR2J3 | M2OR |
| 222 | alpha-Terpineol | OR10A5 | M2OR |
| 223 | alpha-Terpineol | OR5P3 | M2OR |
| 224 | alpha-Pinene | OR2A7 | M2OR |
| 225 | alpha-Pinene | OR2T4 | M2OR |

**Table S9.** Chemical odorant-hOR pairs from this paper and databases.

| **No.** | **Odorant** | **OR** | **Source** |
| --- | --- | --- | --- |
| 1 | Citronellal | OR1A1 | this paper; M2OR |
| 2 | Citronellal | OR1A2 | M2OR |
| 3 | Citronellal | OR1D2 | M2OR |
| 4 | Citronellal | OR2J3 | this paper |
| 5 | Citronellal | OR5K1 | this paper |
| 6 | Eucalyptol | OR10G7 | M2OR |
| 7 | Eucalyptol | OR10J3 | M2OR |
| 8 | Eucalyptol | OR2J3 | M2OR |
| 9 | Eucalyptol | OR2W1 | M2OR |
| 10 | Eucalyptol | OR51B4 | M2OR |
| 11 | Eugenol | OR10G3 | OlfactionBase |
| 12 | Eugenol | OR10G4 | this paper |
| 13 | Eugenol | OR10G7 | this paper |
| 14 | Eugenol | OR14A16 | OlfactionBase |
| 15 | Eugenol | OR1A1 | this paper |
| 16 | Eugenol | OR2A25 | OlfactionBase |
| 17 | Eugenol | OR2C1 | this paper |
| 18 | Eugenol | OR2J3 | this paper |
| 19 | Eugenol | OR2T1 | OlfactionBase |
| 20 | Eugenol | OR2T6 | this paper |
| 21 | Eugenol | OR51E2 | OlfactionBase |
| 22 | Eugenol | OR52B6 | OlfactionBase |
| 23 | Phenylethyl alcohol | OR11H4 | this paper |
| 24 | Phenylethyl alcohol | OR2J3 | this paper |
| 25 | Phenylethyl alcohol | OR2W1 | M2OR |

**Table S10.** Sequence identity for the 7 target hORs.

| OR | Template | Template PDB ID | Sequence identity (%) |
| --- | --- | --- | --- |
| OR2W1 | consOR2 | 8UY0 | 52.5 % |
| OR2C1 | consOR2 | 8UY0 | 56.7 % |
| OR2M3 | consOR2 | 8UY0 | 60.4 % |
| OR2T11 | consOR2 | 8UY0 | 63.1 % |
| OR7A17 | consOR1 | 8UXY | 64.1 % |
| OR10X1 | bmOR6A2 | 9LE0 | 47.4 % |
| OR52L1 | consOR52 | 8HTI | 61.1 % |

**Table S11.** Stereochemical validation of the constructed OR homology models.

| Indicators | OR2M3 | OR2T11 | OR52L1 | OR2C1 | OR2W1 | OR7A17 | OR10X1 |
| --- | --- | --- | --- | --- | --- | --- | --- |
| MolProbity Score | 0.63 | 0.98 | 1.14 | 1.05 | 1.24 | 0.94 | 1.45 |
| Clash Score | 0 | 0.85 | 1.48 | 1.69 | 3.75 | 0.66 | 3.54 |
| Ramachandran Favored | 96.97 % | 97.64 % | 96.28 % | 97.27 % | 97.63 % | 96.56 % | 96.00 % |
| Ramachandran Outliers | 0.00 % | 0.00 % | 0.00 % | 0.34 % | 0.00 % | 0.00 % | 0.67 % |
| Rotamer Outliers | 0.76 % | 1.53 % | 0.40 % | 0.78 % | 0.75 % | 0.39 % | 1.12 % |
| C_β_ Deviations | 0.35 % | 0.00 % | 0.00 % | 0.00 % | 0.00 % | 0.36 % | 0.35 % |
| Bond length outliers | 0.00 % | 0.00 % | 0.04 % | 0.04 % | 0.04 % | 0.04 % | 0.08 % |
| Bond angle outliers | 0.49 % | 0.62 % | 0.81 % | 0.28 % | 0.52 % | 0.54 % | 0.58 % |
| Cis Prolines | 0.00 % | 0.00 % | 0.00 % | 0.00 % | 0.00 % | 0.00 % | 0.33 % |

**Table S12** Subgroup analysis of olfactory function improvements by etiology.

| **Etiology** | **Group** | **n** | **OIT change** | **VAS change** | **QOD change** |
| --- | --- | --- | --- | --- | --- |
| Post-infectious | Herb | 22 | +10.45 | -1.41 | -4.95 |
| Post-infectious | Control | 26 | +5.62 | -1.08 | -2.92 |
| Age-related/neurodegenerative | Herb | 10 | +10.30 | -1.30 | -2.90 |
| Age-related/neurodegenerative | Control | 10 | +6.70 | -0.80 | -3.40 |
| Post-traumatic | Herb | 4 | +18.50 | -1.25 | -1.75 |
| Post-traumatic | Control | 1 | +17.00 | 0.00 | -3.00 |

**Table S13.** Main molecular docking interactions between (-)-Borneol and 7 hORs. Residues in the transmembrane domain were labeled using the Ballesteros-Weinstein numbering system, with residues without a specific numerical index representing residues located in the extracellular loop 2 (ECL2) region.

| OR | Interacting residues (within 3Å) |
| --- | --- |
| OR2W1 | TYR104^3×32^, MET105^3×33^, GLY108^3×36^, SER109^3×37^, ILE206^5×46^, ILE255^6×51^, TYR259^6×55^, TYR278^7×40^ |
| OR2C1 | LEU105^3×33^, GLY108^3×36^, ALA109^3×37^, CYS112^3×40^, ASN155^4×56^, GLN159^4×60^, VAL202^5×42^, CYS203^5×43^, PHE206^5×46^, THR207^5×47^, ALA255^6×51^, TYR259^6×55^ |
| OR2M3 | THR105^3×33^, LEU108^3×36^, ASP159^4×60^, PHE181, LEU199^5×39^, CYS202^5×42^, CYS203^5×43^, MET206^5×46^, ILE207^5×47^, TYR259^6×55^ |
| OR2T11 | LEU102^3×33^, ILE105^3×36^, PHE109^3×40^, ASP152^4×56^, LEU156^4×60^, CYS199^5×42^, CYS200^5×43^, MET203^5×46^, TYR256^6×55^ |
| OR7A17 | VAL105^3×33^, GLY108^3×36^, GLY109^3×37^, GLN159^4×60^, ALA203^5×43^, LEU206^5×46^, ALA207^5×47^ |
| OR10X1 | LEU121^3×33^, GLY124^3×36^, GLY125^3×37^, CYS128^3×40^, ILE171^4×56^, Glu175^4×60^, MET197, ILE218^5×42^, SER219^5×43^, GLY222^5×46^, LEU223^5×47^, TYR275^6×55^ |
| OR52L1 | HIS123^3×33^, LEU174^4×57^, PHE177^4×60^, HIS199, MET200, GLY217^5×39^, MET220^5×42^, ALA221^5×43^, ILE225^5×47^ |

**Table S14.** MM/GBSA Binding Energy Components from Triplicate MD Simulations (mean ± SD).

|  | OR2M3 | OR2T11 | OR52L1 |
| --- | --- | --- | --- |
| VDWAALS | -27.77 ± 0.80 | -25.74 ± 0.66 | -25.47 ± 0.95 |
| EEL | -6.20 ± 1.81 | -6.83 ± 1.71 | -3.48 ± 1.41 |
| EGB | 10.06 ± 1.87 | 11.53 ± 1.18 | 11.10 ± 0.28 |
| ESURF | -3.40 ± 0.14 | -3.36 ± 0.09 | -3.16 ± 0.10 |
| ∆G_gas | -33.97 ± 1.01 | -32.57 ± 2.37 | -28.95 ± 0.80 |
| ∆G_solv | 6.66 ± 1.97 | 8.17 ± 1.09 | 7.94 ± 0.30 |
| ∆TOTAL | -27.31 ± 1.04 | -24.40 ± 1.28 | -21.01 ± 1.08 |
